# Sustainable green concrete: multi-criteria optimization of bentonite–fly ash–silica fume ternary systems integrating life cycle assessment and mechanical performance analysis

**DOI:** 10.1038/s41598-026-59295-z

**Published:** 2026-06-29

**Authors:** Ghadeer Emad, Ahmed Elgamal, Nehal M. Ashoor

**Affiliations:** 1 Department of Civil Engineering, Faculty of Engineering, Port Said University, Port Fuad, Egypt; 2 Department of Civil Engineering, Faculty of Engineering, Damietta University, New Damietta City, Egypt

**Keywords:** Green concrete, Supplementary cementitious materials, Silica fume, Fly ash, Bentonite, Compressive strength, Sulfate resistance, Life cycle assessment, Pareto optimization, Engineering, Environmental sciences, Materials science

## Abstract

Global CO₂ emissions from the cement sector account for approximately 8% of anthropogenic greenhouse gas emissions, underscoring the urgent need for sustainable concrete alternatives based on supplementary cementitious materials (SCMs). This study evaluates the mechanical performance, durability, and environmental impacts of green concrete mixtures incorporating bentonite, either alone or in combination with fly ash and silica fume as partial replacements for ordinary Portland cement (OPC). An experimental program encompassing 22 mixtures assessed workability, compressive strength at multiple ages, and resistance to seawater sulfate attack. A comprehensive life cycle assessment (LCA) was conducted using One Click LCA(tm) software to quantify environmental–mechanical trade-offs, with a case study application to the Damietta University Hospital project in Egypt. The results demonstrate that cement contributes 83–95% of the global warming potential (GWP) across all mixtures, confirming it as the dominant driver of environmental impact. Mixtures containing 50% fly ash achieved a GWP reduction of up to 50.1%. Mix 20 (4% bentonite + 15% silica fume) exhibited the highest compressive strength (49.0 MPa at 28 days, 54.27 MPa at 56 days) and superior seawater sulfate resistance, emerging as the structural optimum. In contrast, Mix 12 delivered the best environmental performance. Crucially, no single mixture simultaneously optimized both mechanical and environmental indicators, underscoring the need for context-dependent mixture selection guided by the Pareto frontier framework developed in this study.

## Introduction

Concrete is the most widely used construction material worldwide, and the second-most consumed substance globally after water. Annual production exceeds 13 billion tons, making it an indispensable component of modern infrastructure and the urban built environment^1^. However, large-scale concrete production entails significant environmental consequences, of which Portland cement manufacturing is the most critical. Cement production is estimated to generate approximately 8% of global CO₂ emissions, primarily from clinker production and limestone calcination^2^. In Egypt specifically, greenhouse gas emissions from the building sector rose by approximately 31% between 2005 and 2015^3^.

The construction industry is also a major consumer of natural resources and energy. The sector accounts for roughly 32% of the world’s natural resources, approximately 40% of global energy consumption, and about one-third of worldwide greenhouse gas emissions^4^. These environmental pressures have motivated extensive research into sustainable construction materials, including the development of eco-friendly alternatives and improvements in energy efficiency in building practices.

Among the most promising strategies for reducing concrete’s environmental footprint is the partial replacement of Portland cement with supplementary cementitious materials (SCMs). Recent studies have further demonstrated the potential of a wide range of supplementary cementitious materials (SCMs) to improve the sustainability and performance of concrete. Metakaolin and rice husk ash have been reported to enhance mechanical properties and durability while reducing cement consumption^5,6^. Sustainable ternary blended systems incorporating metakaolin and rice husk ash have also shown improved strength and resistance to aggressive environments, highlighting the benefits of combining multiple SCMs within a single binder system^7^. In addition, ground granulated blast furnace slag and recycled industrial byproducts have demonstrated favorable durability and environmental performance, supporting the broader transition toward low-carbon concrete technologies^8^.

SCMs exhibit pozzolanic or hydraulic reactivity and can enhance both the strength and durability of concrete^9^. Fly ash, silica fume, and natural pozzolanic materials have been widely investigated for their capacity to reduce cement consumption and the associated CO₂ emissions while improving concrete performance^10,11^.

**Fig. 1:**
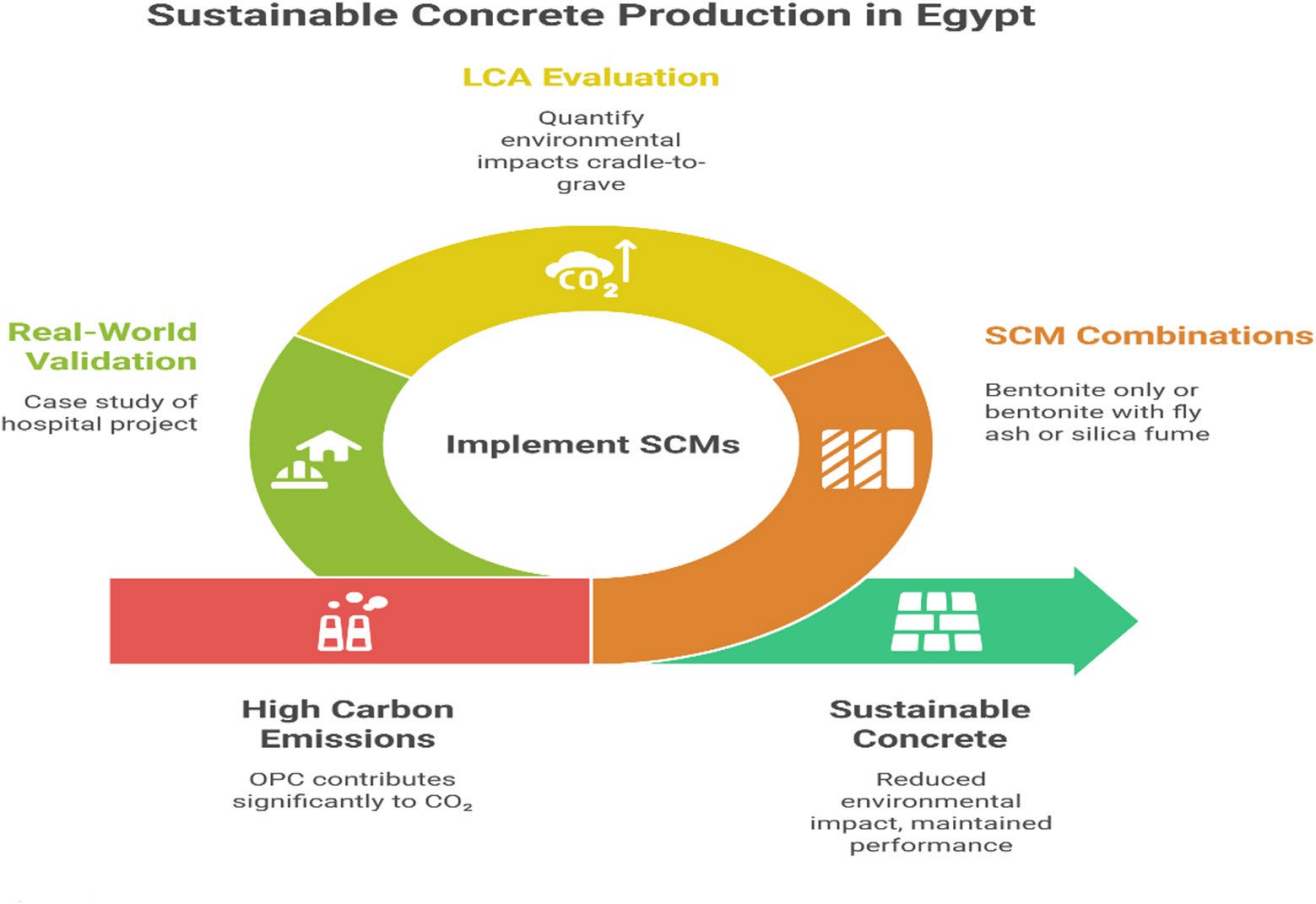
Research framework.

Although several supplementary cementitious materials (SCMs), including ground granulated blast furnace slag (GGBS), metakaolin, rice husk ash, and other industrial byproducts, have demonstrated promising performance in sustainable concrete applications^5–8,12^, the present study focuses on bentonite, fly ash, and silica fume due to their complementary physical and chemical characteristics, as well as their relevance to local construction practices. Bentonite was selected because of its abundance, low cost, and growing potential as a natural SCM capable of improving particle packing and long-term performance^13–15^. Fly ash provides long-term pozzolanic activity and helps reduce cement consumption, while silica fume offers high early-age reactivity, microfiller effects, and substantial pore refinement. Furthermore, the combination of these three materials enables the investigation of synergistic interactions among SCMs with distinctly different particle-size distributions and reactivity levels. Such characteristics make this ternary system particularly suitable for evaluating concrete performance under aggressive marine and sulfate-rich exposure conditions, which are of significant importance for sustainable infrastructure development in Egypt (Fig. 1).

### Nomenclature

A nomenclature table summarizing all symbols, abbreviations, and environmental indicators is provided to enhance clarity and consistency throughout the manuscript.

**Table:** 

Symbol / Abbreviation	Meaning
OPC	Ordinary Portland Cement
SCM	Supplementary Cementitious Material
BN	Bentonite
FA	Fly Ash
SF	Silica Fume
RHA	Rice Husk Ash
MK	Metakaolin
GGBS	Ground Granulated Blast Furnace Slag
RSS	Recycled Steel Slag
ITZ	Interfacial Transition Zone
LCA	Life Cycle Assessment
SEM	Scanning Electron Microscopy
XRD	X-ray Diffraction
C–S–H	Calcium Silicate Hydrate
Ca(OH)₂	Calcium Hydroxide
GWP	Global Warming Potential
ODP	Ozone Depletion Potential
AP	Acidification Potential
EP	Eutrophication Potential
POCP	Photochemical Ozone Creation Potential

### Research gap

Although binary SCM systems have been extensively studied^16,17^, research on ternary blends incorporating bentonite in combination with fly ash and silica fume remains limited. The complementary particle size distributions of bentonite, fly ash, and silica fume offer potential for enhanced particle packing density and microstructural refinement^18^, while their combined pozzolanic activity may improve both early-age and long-term mechanical properties^19^. Furthermore, the performance of bentonite-based systems under sulfate attack in marine environments has not been adequately characterized^20^.

Previous studies, such as Benli (2019), have primarily focused on conventional fly ash–silica fume systems and evaluated mechanical properties, including compressive and flexural strengths and water absorption. However, these investigations have not sufficiently incorporated naturally abundant, locally available materials, such as bentonite, as a key constituent in ternary SCM systems to enhance durability in aggressive marine and sulfate-rich environments. Bentonite has attracted increasing attention as a promising SCM owing to its filler effect and delayed pozzolanic reactivity, which contribute to denser particle packing and sustained long-term strength development (Mekhnache et al.,^13^. Recent research has confirmed that thermally activated bentonite can exhibit significant pozzolanic activity and may replace up to 20–30% of Portland cement while maintaining or improving compressive strength, depending on its mineralogical characteristics and activation conditions^15^. Prior work has also reported that bentonite incorporation can improve sulfate resistance, reduce permeability, and enhance durability in aggressive marine environments, thereby supporting more sustainable construction practices by reducing cement demand, lowering associated CO₂ emissions, and decreasing lifecycle maintenance costs^21^. Nevertheless, most existing studies have remained confined to laboratory-based mechanical evaluations, without integrating comprehensive lifecycle assessment (LCA) methodologies that balance performance gains with environmental burdens.

Although several recent studies have investigated sustainable concrete incorporating metakaolin, rice husk ash, manufactured sand, and other SCMs^5,6,8^), these investigations have primarily focused on mechanical performance and selected durability indicators. Comprehensive frameworks integrating mechanical properties, marine durability assessment, life cycle assessment (LCA), and multi-criteria optimization remain relatively scarce, particularly for bentonite-based ternary blended systems.

A further limitation in the literature is the tendency to evaluate mechanical properties and environmental impacts in isolation^22,23^. Synergistic assessment frameworks that concurrently integrate mechanical characterization, durability testing, LCA, and multi-criteria statistical analysis—including Pearson correlation, heatmap normalization, and Pareto frontier optimization—remain scarce^24,25^.

### Research novelty

This study addresses these gaps by investigating concrete mixtures incorporating bentonite as a supplementary cementitious material, used either alone or in combination with fly ash or silica fume as partial replacements for ordinary Portland cement (OPC). While previous research has explored integrated approaches combining mechanical performance, durability assessment, life cycle assessment (LCA), and optimization techniques, these studies remain inconsistent for ternary blended systems incorporating bentonite, fly ash, and silica fume. In particular, systematic investigations of bentonite-based binary and ternary combinations within a single experimental, durability, and environmental assessment framework remain limited. Moreover, the combined performance of these systems under simultaneous marine and sulfate-rich exposure conditions remains insufficiently investigated, especially in the context of Egyptian construction materials and environmental conditions.

The unique scientific contributions of this study are summarized as follows:

First, the study presents a comprehensive experimental program covering 22 concrete mixtures. Bentonite was used at low replacement levels of 2%, 4%, and 6%. It was combined with fly ash at 20%, 30%, and 50%, and with silica fume at 5%, 10%, and 15%. It enables a clear, systematic comparison of different SCM systems under controlled conditions.

Second, the study links mechanical performance with durability behavior in a unified interpretation. It evaluates compressive strength, splitting tensile strength, and flexural strength, as well as sulfate resistance under seawater exposure and chloride-ion permeability. This provides a consistent view of both strength development and long-term durability.

Third, the study reveals apparent sensitivity in ozone-depletion potential (ODP) results for mixtures containing silica fume. This behavior is influenced by the allocation rules and background datasets used in life cycle assessment (LCA) databases, underscoring an important methodological consideration in the environmental evaluation of byproduct materials.

Fourth, the study translates the experimental and environmental results into a practical decision-support framework. It combines statistical analysis, environmental normalization, and Pareto optimization to support material selection under conflicting mechanical and environmental requirements.

Overall, the study integrates experimental testing, durability assessment, and environmental evaluation to provide practical guidance for sustainable concrete design, with specific relevance to Egyptian infrastructure conditions.

### Research objectives

This study addresses the identified research gaps through the following objectives:

- To evaluate which SCM combinations achieve the greatest reductions across key environmental impact categories.
- To compare the mechanical performance of binary and ternary SCM replacement systems.
- To apply statistical methods—including correlation analysis, clustering, and Pareto optimization—to quantify and navigate the environmental–mechanical trade-off space.
- To derive practical, context-specific guidance for sustainable concrete selection within the Egyptian infrastructure sector.

## Materials and methods

**Fig. 2:**
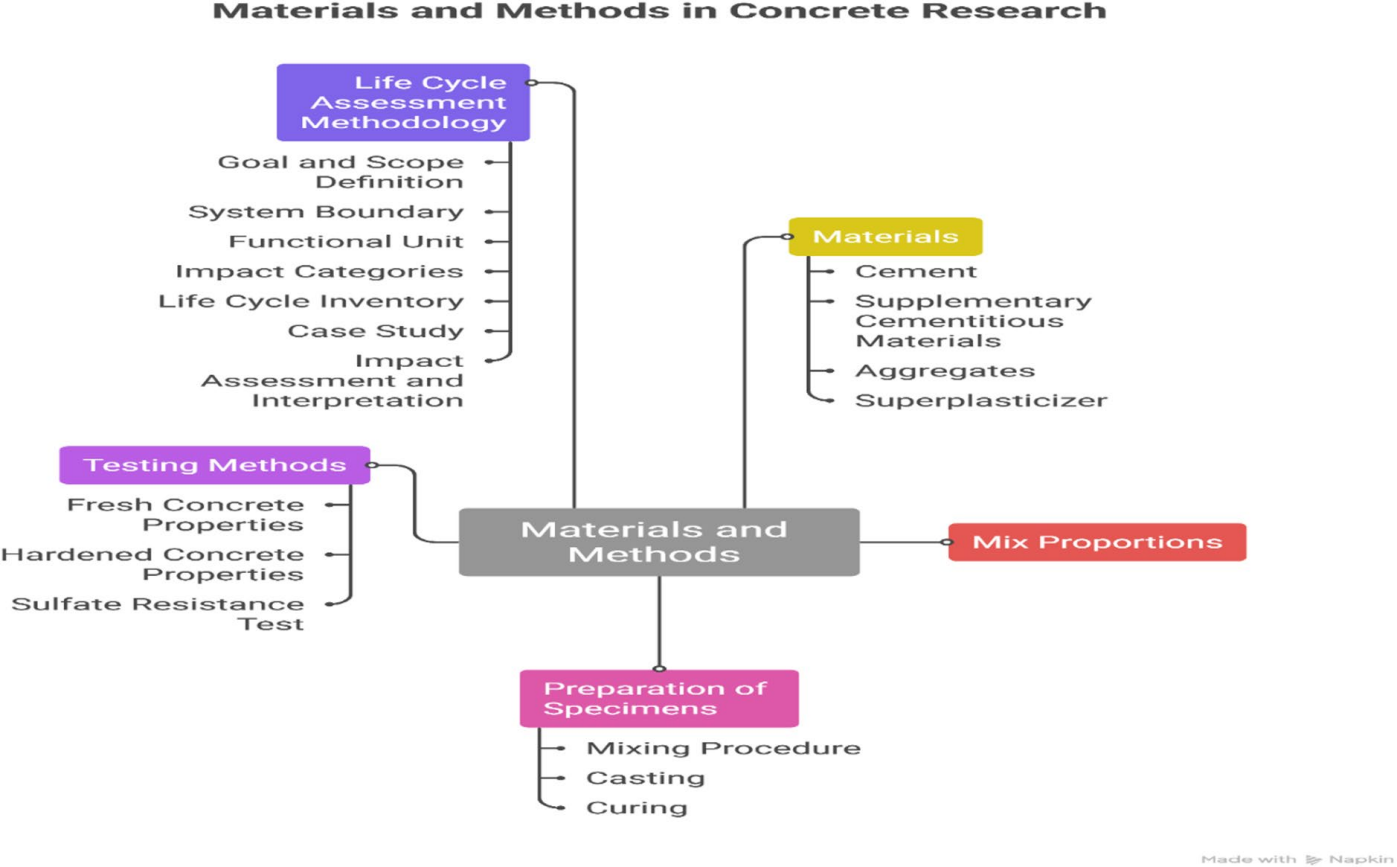
Overview of the experimental program and life cycle assessment framework adopted in this study.

The overall research framework integrates materials characterization, experimental procedures, and life cycle assessment to enable a comprehensive evaluation of green concrete mixtures (Fig. 2).

### Materials

#### Cement

All concrete mixtures were prepared using Ordinary Portland Cement (OPC), type CEM I 42.5 N. This cement type, used as the sole binder in the control mixture, is the most widely available grade in the Egyptian local market and complies with Egyptian Standard Specification ES 4756-1/2013. The cement has a specific gravity of 3.15 and a Blaine fineness of 325 m^2^/kg.

#### Supplementary cementitious materials

Three supplementary cementitious materials (SCMs) were used as partial replacements of OPC to investigate their individual and synergistic effects on concrete performance:

Silica fume (SF): Sika Fume(r)-HR, a byproduct of ferrosilicon alloy production, consists of ultra-fine spherical particles (0.1–0.3 μm) with a high amorphous silica content (> 85%). It has a specific gravity of 2.20 and a surface area of approximately 20,000 m^2^/kg.

Fly ash (FA): Sika Fly Ash, Class F, classified in accordance with ASTM C618. This material was produced from coal combustion in thermal power stations. It consists of spherical particles with diameters ranging from 10 to 100 μm, a specific gravity of 2.25, and a Blaine fineness of 380 m^2^/kg.

Bentonite (BN): Natural calcium bentonite clay with a high swelling capacity and water retention properties. As a member of the smectite group of clays, it has a specific gravity of 2.45 and a particle-size distribution in which 93% of particles pass the 2 μm sieve.

#### Fine and coarse aggregates

**Fig. 3:**
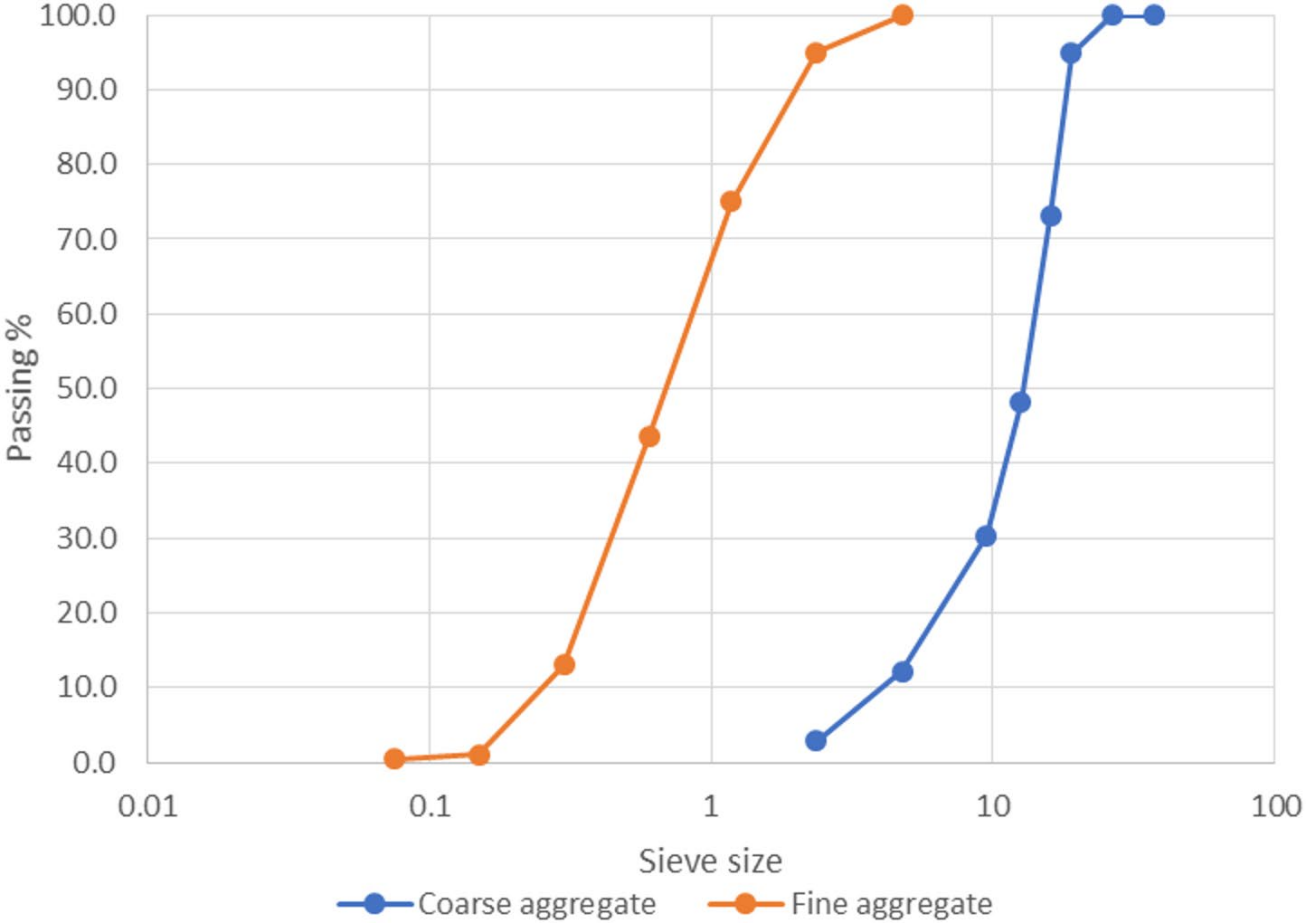
Particle size distribution (grading curves) of fine and coarse aggregates used in this study.

Natural river sand was used as the fine aggregate, and crushed dolomite was used as the coarse aggregate with a nominal maximum size of 19 mm. Both aggregates complied with the Egyptian Standard Specifications for concrete production. The fine aggregate consisted of particles passing the 4.75 mm sieve, while the coarse aggregate was retained on the same sieve. All aggregates were clean, well-graded, and free from deleterious substances. The particle size distribution curves are presented in Fig. 3.

**Fig. 4:**
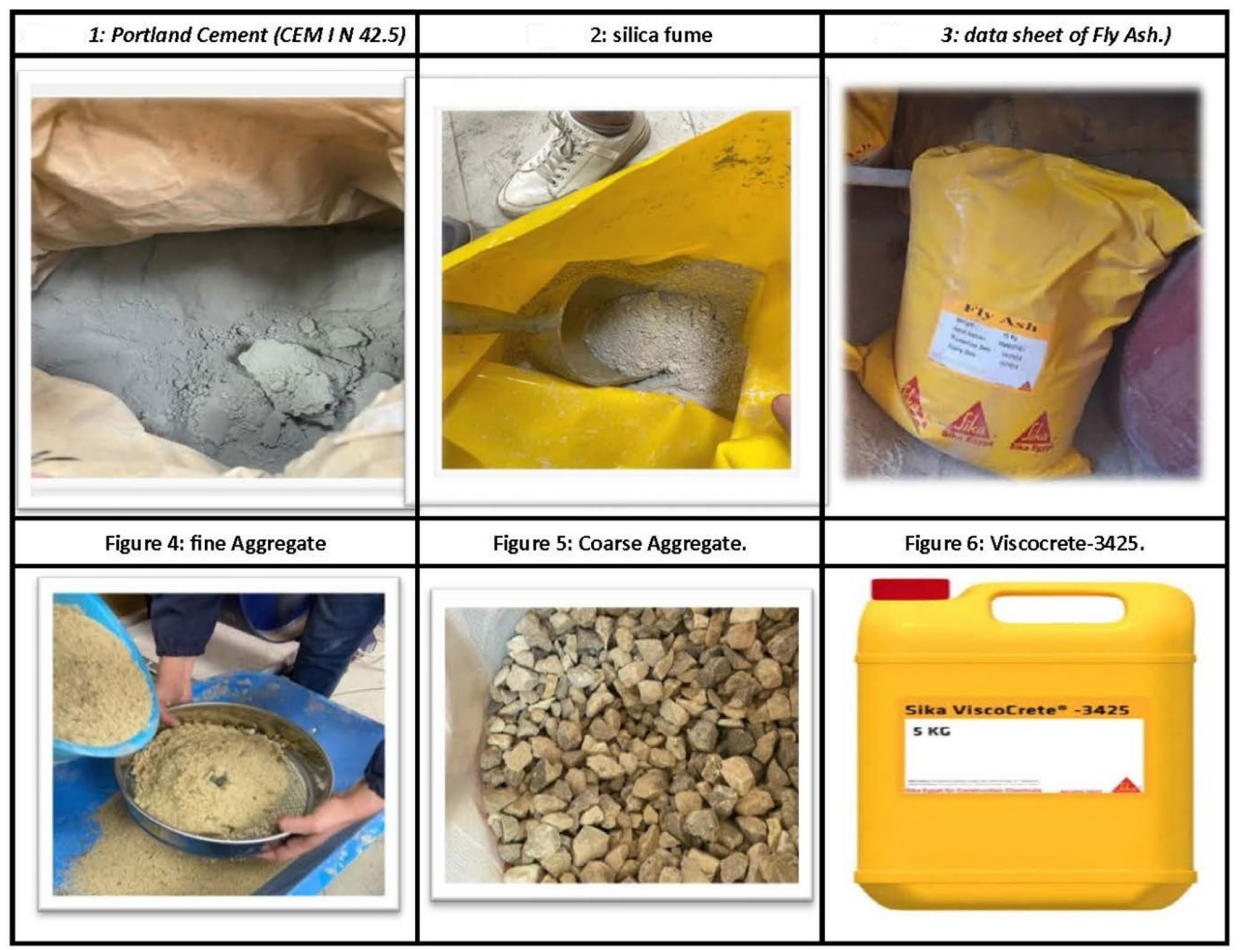
Used materials.

**Table 1:** Chemical composition of cementitious materials (% by mass).

Material	SiO_2_	Al_2_O_3_	Fe_2_O_3_	CaO	MgO	SO_3_	Na_2_O	K_2_O	TiO_2_	*P*_2_O_5_	LOI
OPC	19.12	4.22	3.30	64.69	1.64	2.38	0.21	0.48	0.11	0.08	2.77
FA	63.15	31.11	1.60	0.82	0.90	0.21	0.41	0.52	0.79	0.20	1.29
SF	94.70	0.35	0.28	0.30	0.71	0.47	0.65	0.18	—	0.07	2.29
BN	56.30	24.66	5.89	1.90	2.51	—	1.12	0.48	0.05	0.01	7.08

Note: The high SiO_2_ contents in FA (63.15%), SF (94.70%), and BN (56.30%) confirm their pozzolanic nature.

### Mix proportions

The replacement levels for each SCM were selected following an extensive review of the literature to ensure adequate coverage of potential synergistic effects. Bentonite replacement levels of 2%, 4%, and 6% were adopted based on studies by Akbar et al.^26^ and Waqas et al.^27^. Fly ash replacements of 20%, 30%, and 50% were chosen following the recommendations of Malhotra and Mehta^28^ and Kurda et al.^29^. Silica fume replacements of 5%, 10%, and 15% were selected based on the work of Siddique and Khan^30^ and Aïtcin^31^ (Fig. 4, Table 1).

A total of 22 concrete mixtures were prepared to investigate both the individual and synergistic effects of the three SCMs. The control mixture contained only OPC (Mix C). Mixtures incorporating only bentonite (Mixes 1–3) contained replacement levels of 2%, 4%, and 6%. Two additional series were also prepared: ternary blends containing fly ash (Mixes 4–12) at 20%, 30%, and 50% replacement, and ternary blends containing silica fume (Mixes 13–21) at 5%, 10%, and 15% replacement.

**Fig. 5:**
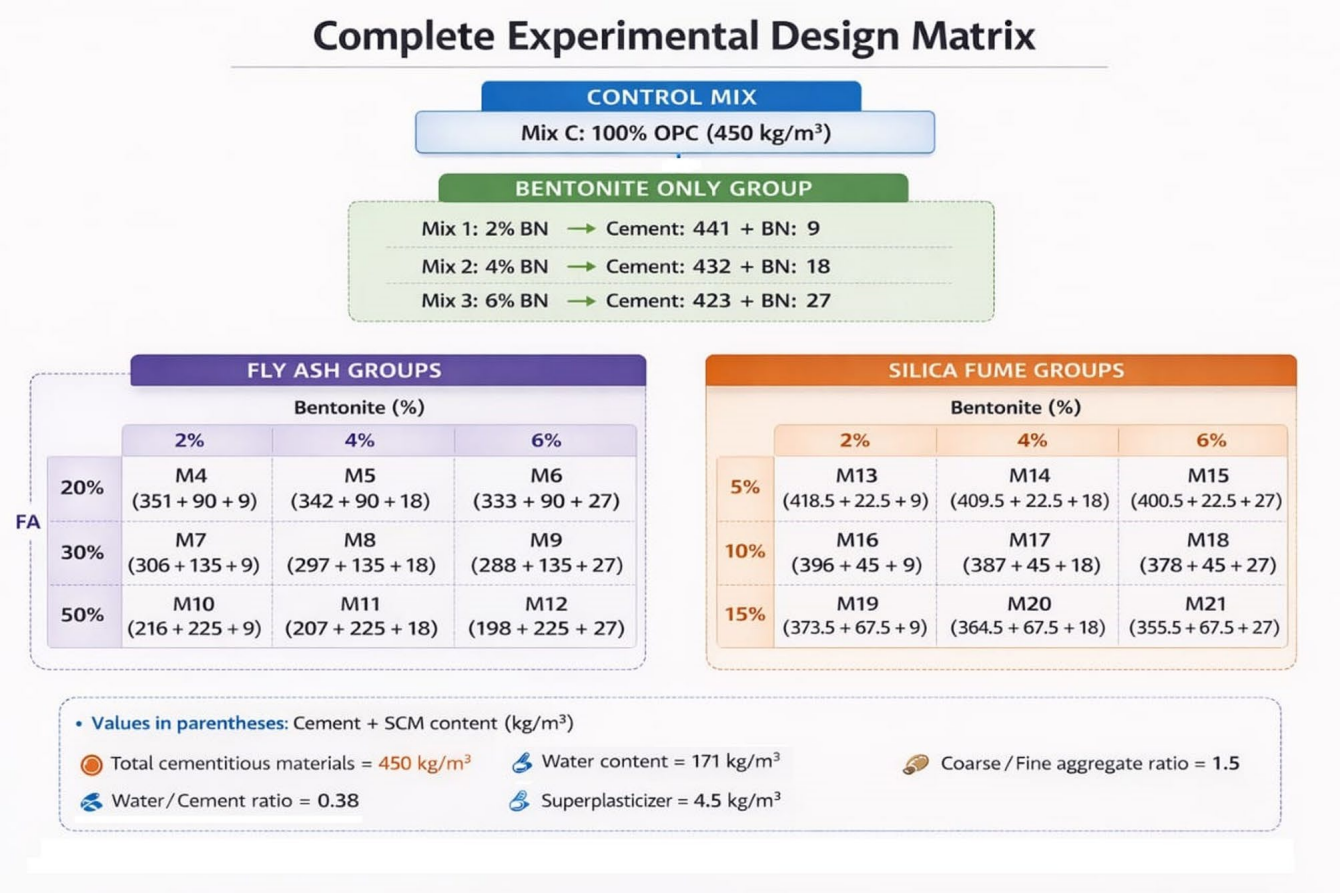
Experimental design matrix illustrating all 22 concrete mixtures investigated in this study.

The total binder content was maintained constant at 450 kg/m³ for all mixtures. Table 2 presents the detailed replacement proportions of bentonite (BN), fly ash (FA), and silica fume (SF), along with the water-to-binder ratio (W/B), aggregate contents, and superplasticizer dosages. A constant W/B ratio of 0.38 was used throughout the experimental program (Fig. 5).

**Table 2:** Detailed mix proportions of all concrete mixtures.

Mix ID	Total binder (kg/m³)	Bentonite (%)	Fly ash (%)	Silica fume (%)	W/B	CA/FA ratio	SP (%)
Control	450	0	0	0	0.38	1.5	1
M1	450	2	0	0	0.38	1.5	1
M2	450	4	0	0	0.38	1.5	1
M3	450	6	0	0	0.38	1.5	1
M4	450	2	20	0	0.38	1.5	1
M5	450	4	20	0	0.38	1.5	1
M6	450	6	20	0	0.38	1.5	1
M7	450	2	30	0	0.38	1.5	1
M8	450	4	30	0	0.38	1.5	1
M9	450	6	30	0	0.38	1.5	1
M10	450	2	50	0	0.38	1.5	1
M11	450	4	50	0	0.38	1.5	1
M12	450	6	50	0	0.38	1.5	1
M13	450	2	0	5	0.38	1.5	1
M14	450	4	0	5	0.38	1.5	1
M15	450	6	0	5	0.38	1.5	1
M16	450	2	0	10	0.38	1.5	1
M17	450	4	0	10	0.38	1.5	1
M18	450	6	0	10	0.38	1.5	1
M19	450	2	0	15	0.38	1.5	1
M20	450	4	0	15	0.38	1.5	1
M21	450	6	0	15	0.38	1.5	1

Note: The water-to-binder ratio (W/B) was fixed at 0.38 for all concrete mixtures. CA = coarse aggregate, FA = fine aggregate, SP = superplasticizer.

### Mixing procedure

#### Mixing sequence

Each mixture component was weighed to the required mass prior to batching. Mixing was performed using a drum mixer. The procedure consisted of two stages. First, fine aggregate (sand) and coarse aggregate (crushed dolomite) were loaded into the mixer and dry-mixed for 5 min to ensure homogeneity. Second, cement and the designated SCMs were added to the aggregate blend, and dry mixing was continued for an additional period to achieve uniform dispersion of the binder constituents.

**Fig. 6:**
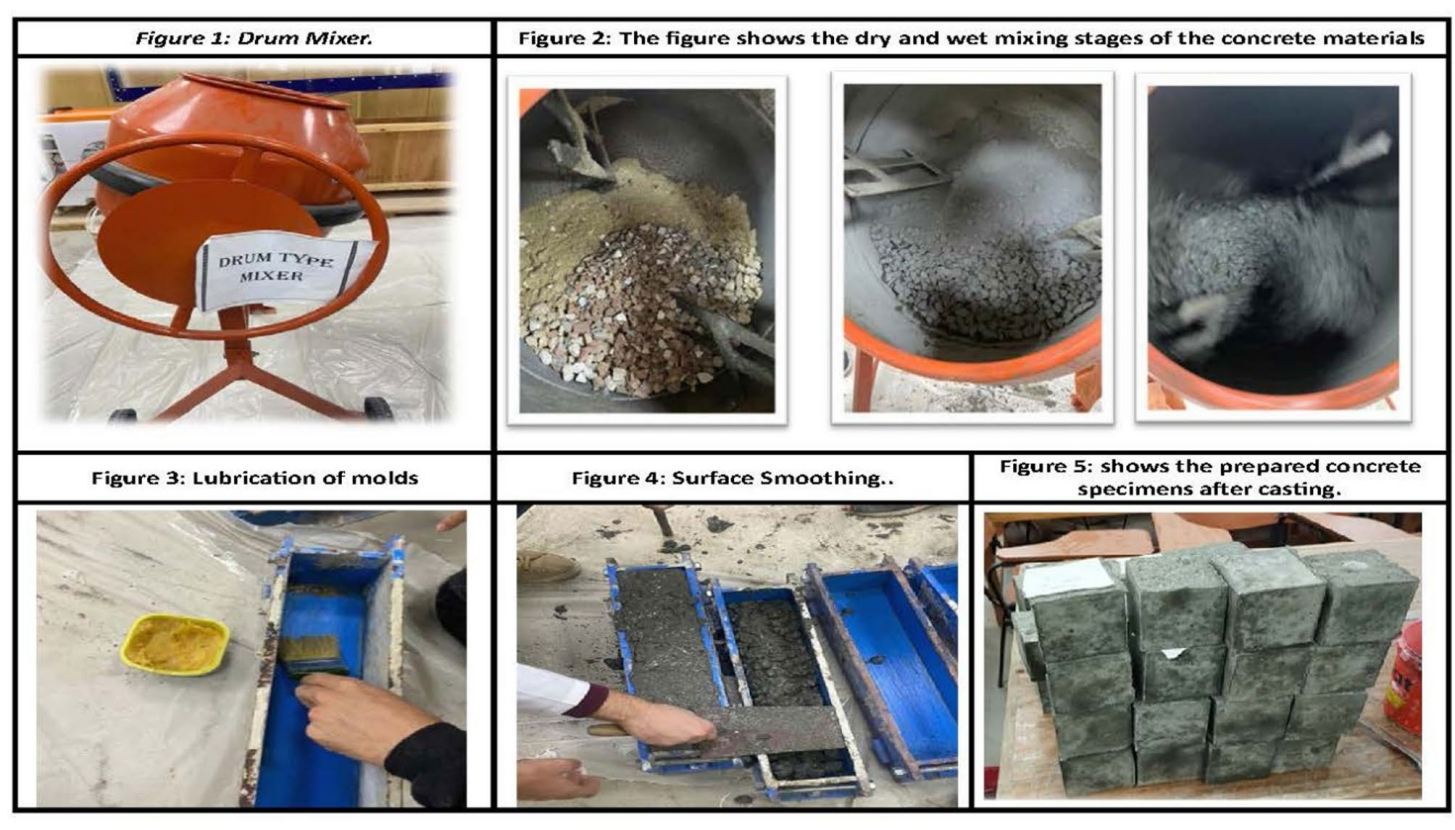
Mixing procedure.

After the dry mixing stage, the mixing water containing the dissolved superplasticizer (Viscocrete-3425) was slowly added to the mixture to ensure adequate workability. Wet mixing was then performed for 3 min to produce a lump-free, homogeneous concrete (Fig. 6).

#### Mixing conditions

Concrete mixing and casting were conducted under controlled laboratory conditions at an ambient temperature of 25 ± 2 °C. For each mixture, the required specimens were prepared for mechanical and durability testing as follows: 18 cubes (100 × 100 × 100 mm) for compressive strength and sulfate resistance, 3 beams (100 × 100 × 500 mm) for flexural strength, 3 cylinders (diameter 150 × 300 mm) for splitting tensile strength, and 4 small cylinders (diameter 100 × 50 mm) for the rapid chloride permeability test (RCPT).

#### Casting

**Fig. 7:**
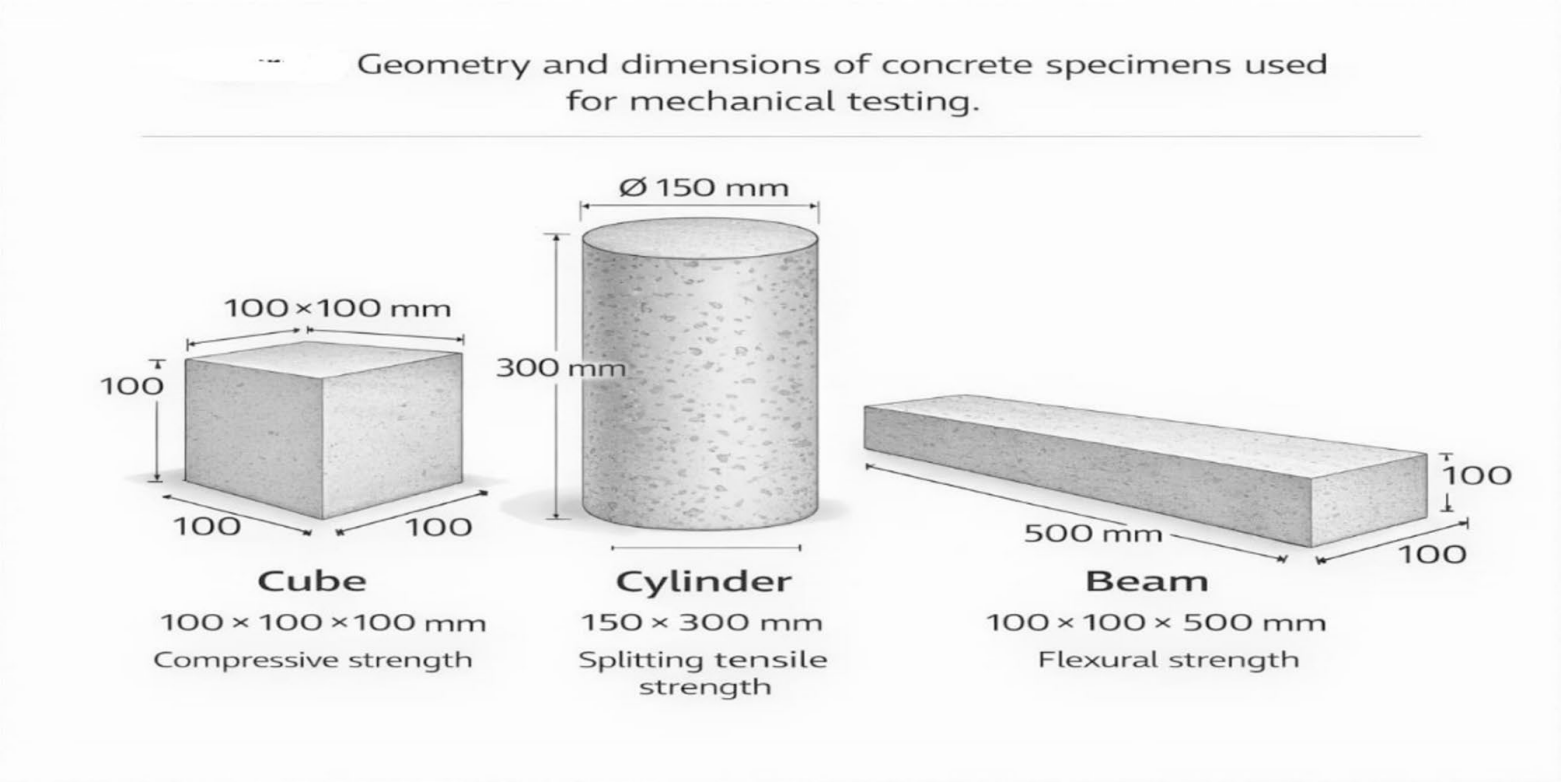
Geometry and dimensions of concrete specimens.

The geometry and dimensions of all concrete specimens used for mechanical testing are illustrated in Fig. 7. The molds were cleaned, coated with a release agent, and filled with fresh concrete in three equal layers. Each layer was compacted by hand-tamping 25 times with a tamping rod to eliminate entrapped air and ensure proper consolidation.

#### Curing

**Fig. 8:**
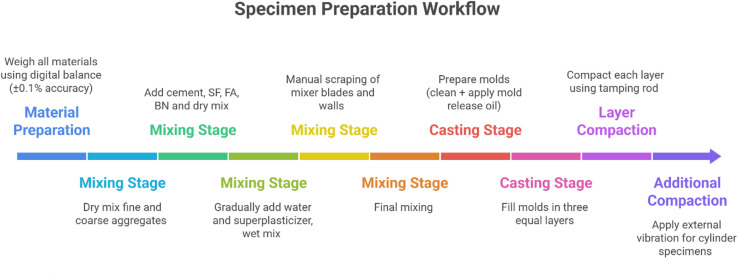
Specimen preparation workflow.

Following casting, all specimens were stored in their molds at laboratory room temperature for 24 h. They were then demolded and transferred to a water-curing tank filled with tap water for continuous moist curing until the designated testing age (Fig. 8).

### Testing methods for fresh and hardened concrete

#### Fresh concrete properties

##### Workability (slump test)

The workability of fresh concrete was evaluated immediately after mixing using the slump cone test in accordance with ASTM C143/C143M-20 and the Egyptian Code of Practice. The measured slump values ranged from 16 to 20 cm.

##### Fresh density

The fresh density of concrete was determined in accordance with ASTM C138/C138M-17a using the slump cone as a temporary volumetric measure.

#### Hardened concrete properties

All mechanical and durability test results are reported as the mean of three replicate specimens to ensure reliability and compliance with standard testing procedures. Specimen preparation, handling, and testing were conducted under controlled laboratory conditions.

##### Compressive strength

Compressive strength was determined using 100 mm cube specimens tested at 7, 28, and 56 days in accordance with ASTM C39/C39M-21 and the Egyptian Code ECP 203–2020. A size correction factor of 0.97 was applied to convert the 100 mm cube strengths to equivalent 150 mm cube values.

##### Splitting tensile strength

Splitting tensile strength was determined on 150 × 300 mm cylinders at 28 days in accordance with ASTM C496/C496M-17.

##### Flexural strength

**Fig. 9:**
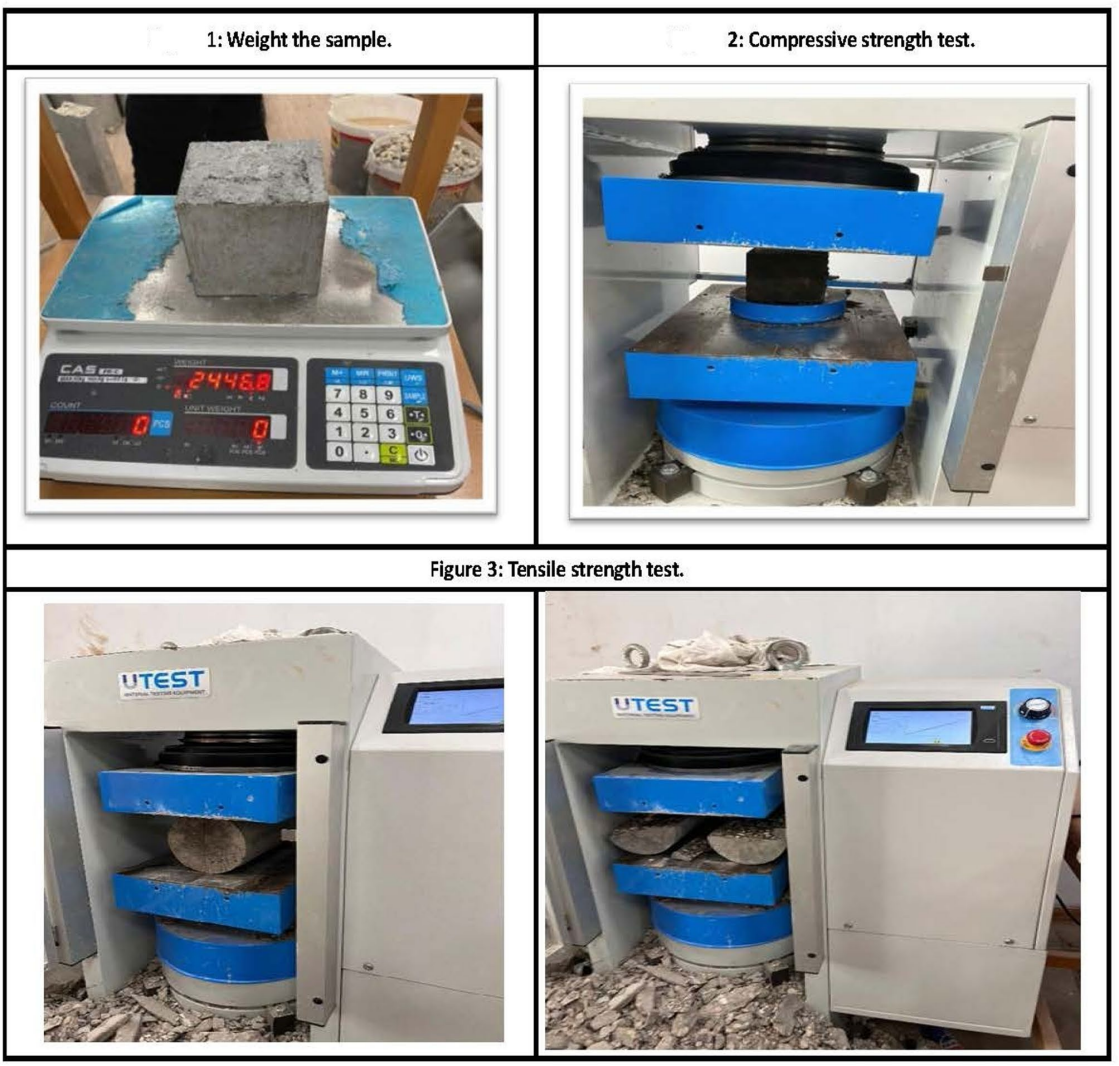
Concrete specimens prepared for testing: weighing the samples, tensile testing setup, and compressive testing setup.

Flexural strength (modulus of rupture) was determined on 100 × 100 × 500 mm prismatic beams at 28 days using four-point loading in accordance with ASTM C78/C78M-22 (Fig. 9).

#### Sulfate resistance test

Sulfate resistance was assessed by immersing concrete cube specimens in either seawater or tap water and monitoring their mass change and compressive strength retention over time. For each mixture, nine cubes were submerged in seawater, and nine in tap water (serving as the control condition). Specimens were tested after 7, 28, and 56 days of exposure to evaluate the degree of deterioration under sulfate attack.

#### Rapid chloride permeability test (RCPT)

**Fig. 10:**
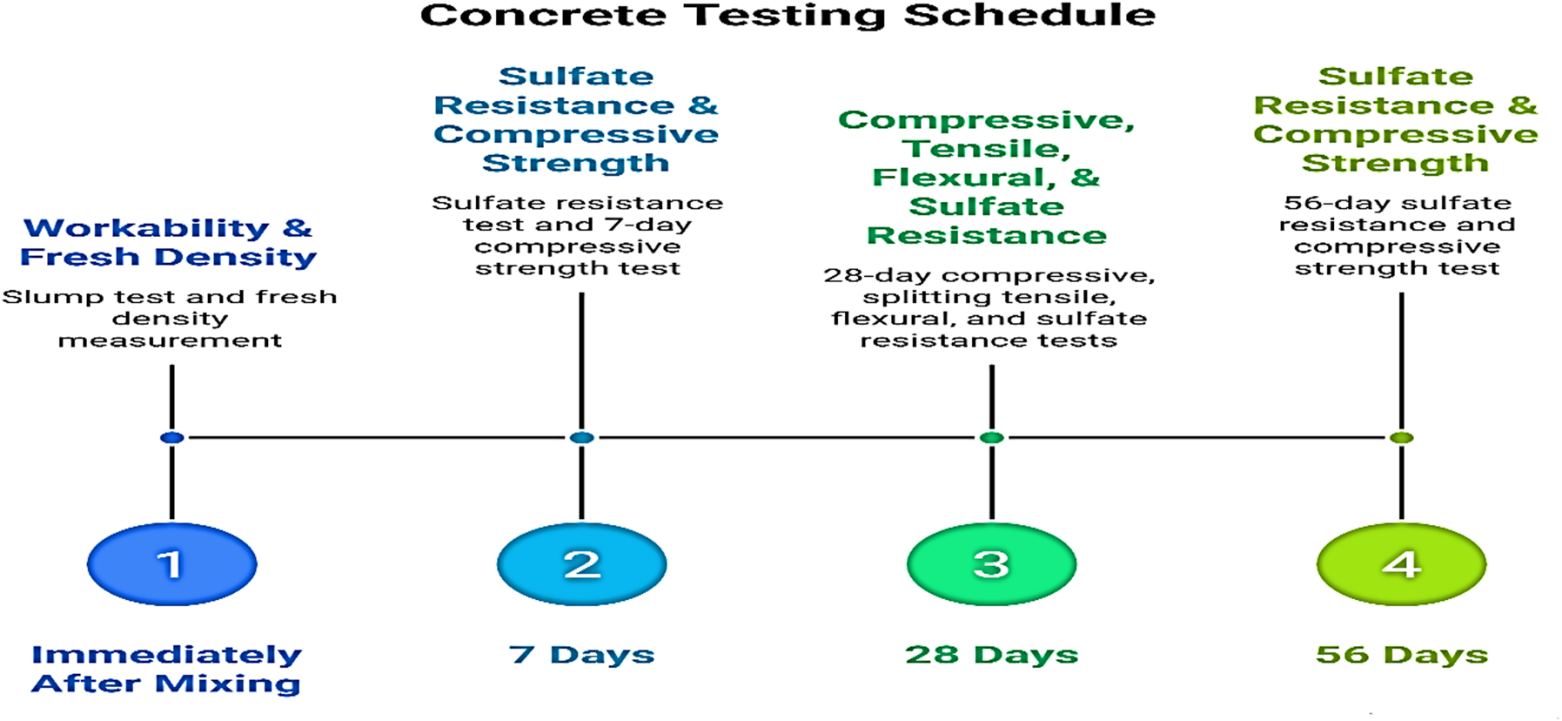
Concrete test schedule.

The resistance to chloride ion ingress was evaluated using the rapid chloride permeability test (RCPT) in accordance with ASTM C1202-22. Testing was conducted at 56 days of age. Cylindrical specimens (100 × 50 mm) were subjected to a 60 V DC electrical potential for 6 h, and the total charge passed (in Coulombs) was recorded as the indicator of chloride penetration resistance (Fig. 10).

### Life cycle assessment (LCA) methodology

#### Goal and scope definition

The LCA methodology adopted in this study conforms to the frameworks established in EN 15978:2011 (assessment of environmental performance of buildings), ISO 14,040, and ISO 14,044 (principles and guidelines for conducting an LCA).

The objective of this analysis was to quantify and compare the environmental impacts of the 22 concrete mixture designs relative to conventional OPC concrete. The assessment was applied to a real-world case study: the New Damietta University Hospital, currently under construction. The LCA was performed using One Click LCA software to enable comparative evaluation of the environmental profiles associated with each cementitious mixture.

##### Functional unit

The functional unit for the whole-building life cycle assessment was defined as 1 m^2^ of gross floor area (GFA) over a 60-year reference study period (RSP), consistent with established whole-building LCA practice and LEED recommendations. Structural concrete quantities for the New Damietta University Hospital were modeled in One Click LCA based on the project’s bill of quantities (BOQ). All 22 concrete mixture scenarios were evaluated against the same building geometry and structural configuration to enable a direct assessment of the environmental–mechanical performance trade-off.

##### Database versions

The One Click LCA cloud platform (January 2025 release, accessed February 2026) was used in this study. Environmental data were obtained primarily from verified Environmental Product Declarations (EPDs) compliant with EN 15,804 + A2 for the Egyptian market. When local EPDs were unavailable, regional generic datasets from One Click LCA (derived from Ecoinvent v3.10) were used. The use of generic datasets is a well-established practice in LCA studies when local data are unavailable^32,33^.

##### Transport assumptions

No supplier-specific transport distances or vehicle types were manually entered. Transportation scenarios were automatically generated by the One Click LCA platform based on the project location (New Damietta University Hospital, Egypt). Default regional assumptions were applied to all materials, including an approximate transport distance of 40 km for sand delivery, based on embedded dumper truck datasets.

##### Energy mix assumptions

Energy mix data for material manufacturing and upstream processes were sourced from verified datasets and EPDs within the One Click LCA database. The Egyptian electricity grid mix (IEA Electricity, Egypt, 2023) was applied automatically through the software’s regionalized database. No additional manual energy scenarios were defined.

##### Allocation procedures

Allocation rules were adopted directly from the selected Environmental Product Declarations (EPDs) and background datasets integrated within One Click LCA. For industrial secondary byproducts such as fly ash and silica fume, a cut-off allocation approach was adopted, consistent with EN 15,804 + A2 recommendations. Under this approach, zero initial environmental burdens are assigned to the byproduct mass prior to subsequent processing and transportation. However, within the background database framework (Ecoinvent v3.10 system model), certain multi-category indicators—such as the ozone depletion potential (ODP)—inherently incorporate upstream economic allocation weights from the primary metallurgical smelting industry, thereby capturing inherited process-specific trace emissions.

#### System boundary

A cradle-to-grave system boundary (excluding the operational use stage, Modules B1–B7) was adopted, as defined in EN 15978:2011. The following life cycle modules were included:

##### Product stage (A1–A3)

Raw material extraction and processing, manufacture of constituent materials (cement, aggregates, and SCMs), and concrete production at the batching plant.

##### Construction stage (A4–A5)

Transportation of concrete from the batching plant to the construction site (Damietta University Hospital) and on-site construction works.

##### Use stage (B1–B7)

Excluded from the scope of this study.

##### End-of-life stage (C1–C4)

Demolition, collection and transport to waste processing facilities, waste processing and recycling, and disposal of residual materials.

#### Life cycle inventory (LCI)

The environmental profiles of the developed concrete mixtures were evaluated using the New Damietta University Hospital as a real-world case study. Total concrete volumes were determined through quantity take-off procedures based on the project’s structural and architectural drawings. These quantities were then entered as inputs into One Click LCA. Table 3 presents a summary of the building-level life cycle inventory and dataset sources for four representative concrete scenarios; the same approach was applied to all 22 mixtures.

**Table 3:** Summarized building-level life cycle inventory (LCI) and dataset sources for selected representative concrete scenarios.

Material	Unit	Control Mix	M1 (2% BN)	M12 (6% BN + 50% FA)	M20 (4% BN + 15% SF)	Dataset / EPD Source
Cement	kg	13,414,535.0	13,146,244.0	5,902,395.0	10,865,773.0	EPD-IES-12620
	kg/m^2^	217.9	213.6	95.9	176.5	
Bentonite	kg	0.0	268,291.0	804,872.0	536,581.0	Generic dataset (One Click LCA)
	kg/m^2^	0.0	4.4	13.1	8.7	
Fly ash	kg	0.0	0.0	6,707,267.0	0.0	Generic dataset (One Click LCA)
	kg/m^2^	0.0	0.0	109.0	0.0	
Silica fume	kg	0.0	0.0	0.0	2,012,180.0	Generic dataset (One Click LCA)
	kg/m^2^	0.0	0.0	0.0	32.7	
Superplasticizer	kg	134,145.0	134,145.0	134,145.0	134,145.0	HUB-4231 (EPD Hub)
	kg/m^2^	2.2	2.2	2.2	2.2	
Sand	kg	21,535,173.0	21,530,946.0	20,584,161.0	21,258,030.0	Generic dataset (One Click LCA)
	kg/m^2^	349.8	349.8	334.4	345.3	
Gravel	kg	32,302,759.0	32,296,420.0	30,876,241.0	31,887,045.0	Generic dataset (One Click LCA)
	kg/m^2^	524.8	524.7	501.6	518.0	
Water	kg	5,097,523.0	5,097,523.0	5,097,523.0	5,097,523.0	EXCLUDED

Note: Values reported in kg/m^2^ represent material quantities normalized per square meter of gross floor area (GFA). Tap water was excluded from the LCA model due to the absence of region-specific Egyptian EPDs or verified generic datasets within the One Click LCA database.

#### Impact categories

**Fig. 11:**
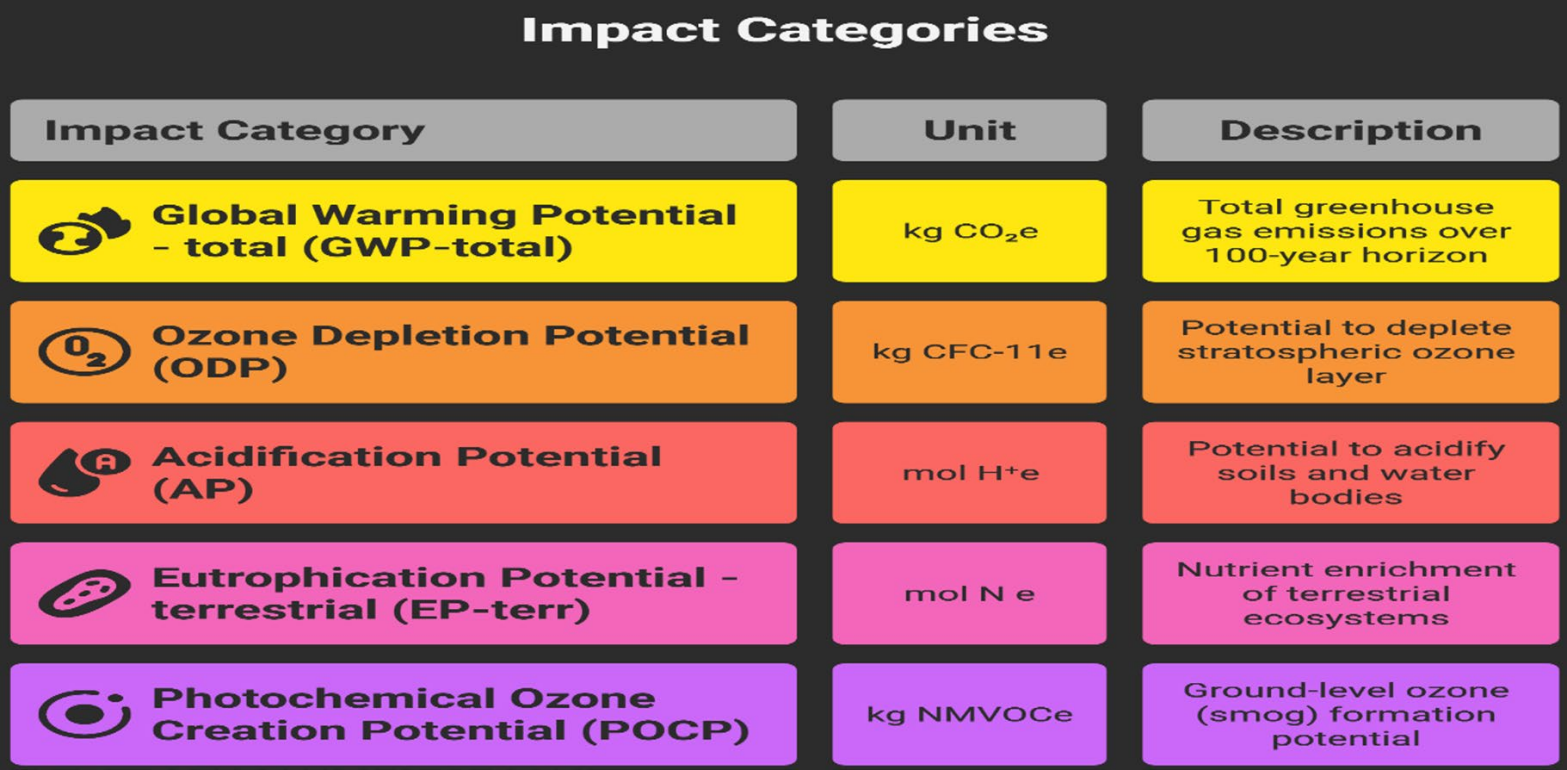
Impact categories evaluated in accordance with EN 15,804 + A2.

The following midpoint impact categories were evaluated as the sustainability core indicators specified in EN 15,804 + A2: global warming potential (GWP), ozone depletion potential (ODP), acidification potential (AP), eutrophication potential (EP), and photochemical ozone creation potential (POCP) (Fig. 11).

### Statistical analysis methods

Statistical analyses were performed to evaluate differences in the mechanical properties among the investigated concrete mixtures. One-way analysis of variance (ANOVA) was used to compare compressive strength (at 28 and 56 days), splitting tensile strength, and flexural strength across the four concrete groups.

Levene’s test was first conducted to verify the assumption of homogeneity of variances prior to performing ANOVA. Where significant differences were identified, Tukey’s honestly significant difference (HSD) post-hoc test was applied to determine pairwise differences between group means.

The coefficient of variation (CV, %) was also calculated to assess data variability and experimental repeatability. Lower CV values indicate greater consistency of the experimental measurements. Statistical significance was accepted at *p* < 0.05.

A Pareto-based multi-criteria analysis was additionally performed to evaluate the trade-off between mechanical performance and environmental impact, using compressive strength and global warming potential (GWP) reduction as the optimization objectives. All statistical analyses were performed using Python 3.x with the NumPy, SciPy, and Matplotlib libraries.

## Results: mechanical performance

### Compressive strength

**Fig. 12:**
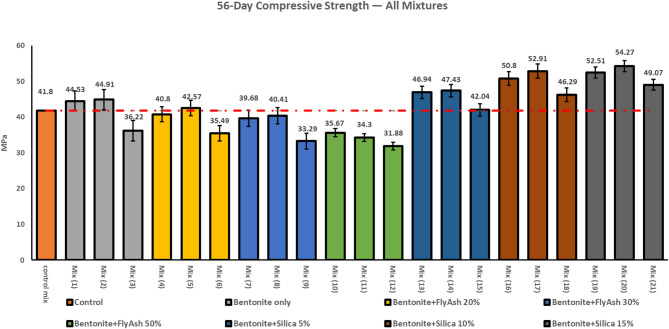
Comparison of 56-day compressive strength for all investigated concrete mixtures, including mean values and error bars. The red dashed line represents the compressive strength of the control mixture.

**Fig. 13:**
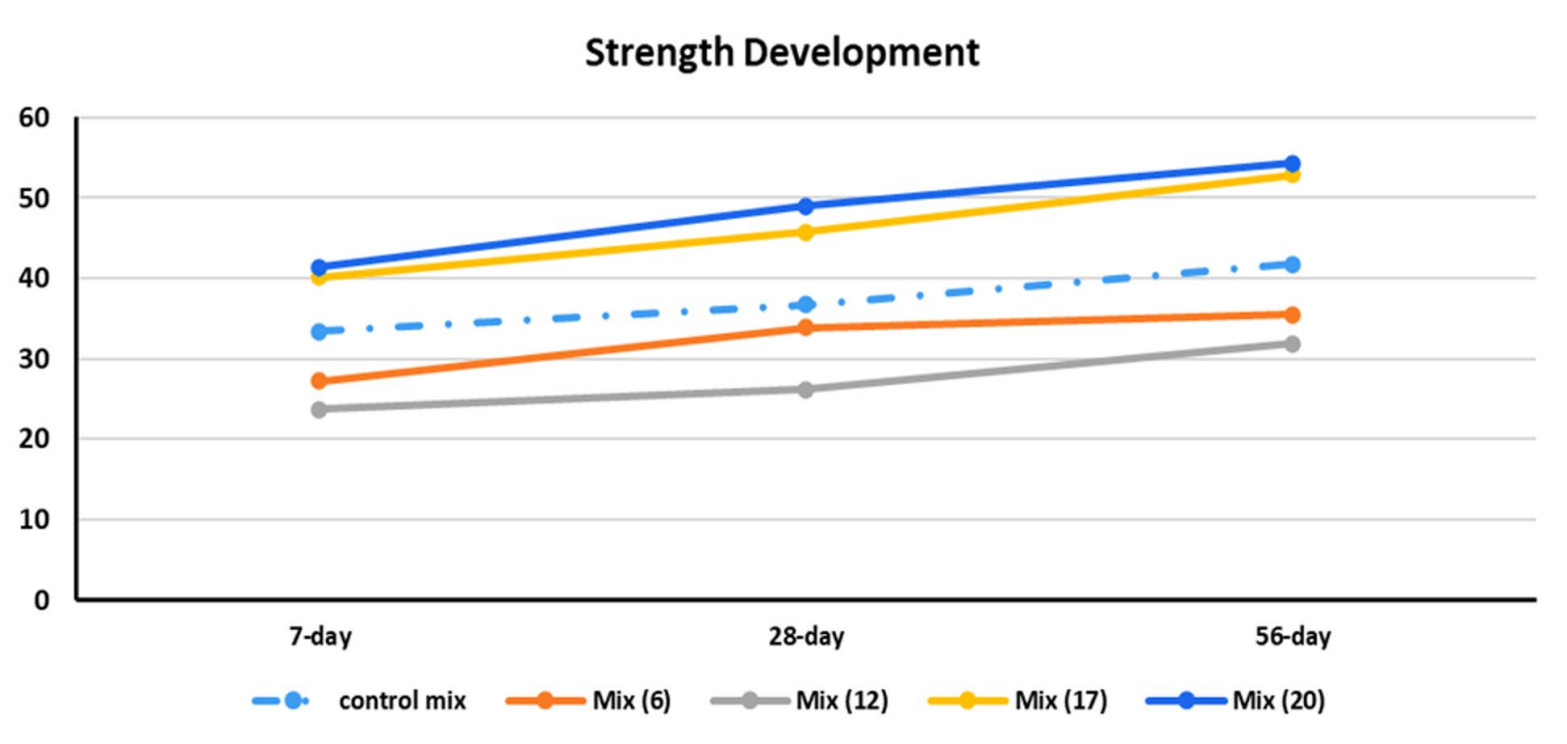
Compressive strength development curves at 7, 28, and 56 days for selected concrete mix designs.

The 56-day compressive strengths of all investigated mixtures are summarized in Fig. 12, and the strength development of selected mixes at 7, 28, and 56 days is presented in Fig. 13. The control mix achieved a 56-day compressive strength of 41.8 MPa, which served as the benchmark for evaluating the performance of bentonite- and SCM-modified mixtures.

Concrete mixes incorporating silica fume (Mixes 16–21) exhibited the highest compressive strengths. At 56 days, the top-performing mixtures were Mix 20 (54.27 MPa), Mix 17 (52.91 MPa), and Mix 19 (52.51 MPa). In contrast, increasing the fly ash content reduced compressive strength relative to the control (Table 4).

**Table 4:** Compressive strength development of selected concrete mixtures at 7, 28, and 56 days.

Mix	Description	7-day (MPa)	28-day (MPa)	56-day (MPa)	Change
Control	OPC reference	33.41	36.76	41.80	—
Mix 6	BN + FA	27.00	34.00	35.49	-15.1%
Mix 12	BN + 50% FA	23.71	26.18	31.88	-23.7%
Mix 17	BN + 10% SF	40.80	46.00	52.91	+ 26.6%
Mix 20	BN + 15% SF	41.30	49.00	54.27	+ 29.8%

A one-way between-groups ANOVA was conducted to compare compressive strength across four concrete groups at 28 and 56 days: Control (100% OPC, *n* = 1), Group I (bentonite only, *n* = 3), Group II (bentonite + fly ash, *n* = 9), and Group III (bentonite + silica fume, *n* = 9). Levene’s test confirmed homogeneity of variances at both ages (28 d: *p* = 0.530; 56 d: *p* = 0.844) (Table 5).

**Table 5:** One-way ANOVA results for 28-day and 56-day compressive strength (between groups).

Age	Source	Sum of Squares	df	Mean Square	F	*p*-value	eta-squared
28 days	Between Groups	587.18	3	195.73	17.94	< 0.001	0.75
56 days	Between Groups	655.29	3	218.43	13.81	< 0.001	0.70

Significant differences were detected among the four groups at both curing ages (28 d: *F* = 17.94, *p* < 0.001; 56 d: *F* = 13.81, *p* < 0.001). The effect sizes were large (eta-squared = 0.75 and 0.70 at 28 and 56 days, respectively), indicating that group membership accounted for approximately 70–75% of the variance in compressive strength (Table 6).

**Table 6:** Tukey HSD homogeneous subsets for 28-day and 56-day compressive strength (between groups).

Age	Group	*N*	Subset for alpha = 0.05	
			A	B
28 days	Group II (Bentonite + Fly Ash)	9	32.59	
	Group I (Bentonite only)	3	36.80	36.80
	Group III (Bentonite + Silica Fume)	9		43.95
	**Sig.**		**0.119**	**1.000**
56 days	Group II (Bentonite + Fly Ash)	9	37.12	
	Group I (Bentonite only)	3	41.89	41.89
	Group III (Bentonite + Silica Fume)	9		49.14
	**Sig.**		**0.149**	**1.000**

Post-hoc comparisons using Tukey HSD showed that Group III (bentonite + silica fume) achieved significantly higher compressive strength than Group II (bentonite + fly ash) at both curing ages (*p* < 0.001). Group III also differed significantly from Group I. No significant difference was observed between Group I and Group II (*p* > 0.05). The homogeneous-subset analysis confirmed that Group III had the highest compressive strength, whereas Group II had the lowest. These results underscore the beneficial effect of silica fume on compressive strength development.

### Strength distribution across groups

**Fig. 14:**
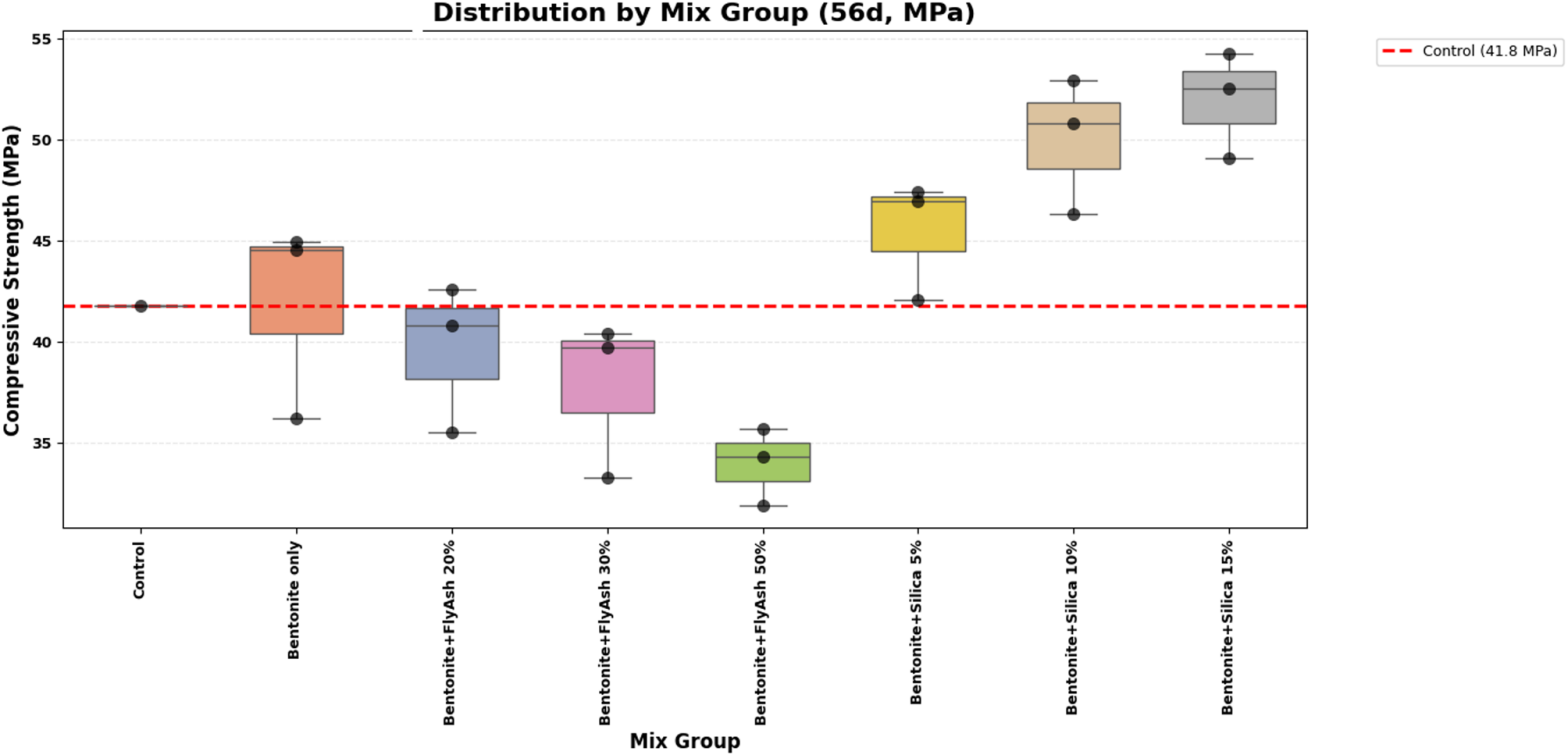
Box-plot distributional analysis of 56-day compressive strength stratified by SCM group.

The compressive strength data showed distinct distributions across the three experimental groups. Group I (bentonite only) produced strengths ranging from approximately 36 to 45 MPa at 56 days, with most values near or slightly below the control. Group II (bentonite + fly ash) showed greater scatter, spanning approximately 32–44 MPa, and increasing fly ash content (20% and 30%) was associated with progressive strength reduction. Group III (bentonite + silica fume) achieved the highest strengths overall, ranging from approximately 42 to 54.27 MPa at 56 days (Fig. 14).

Statistical Comparison of Individual Group III Mixtures (Bentonite + Silica Fume):

A one-way ANOVA was conducted to compare compressive strength among the nine individual mixtures within Group III at 28 and 56 days, with three replicate specimens per mixture (*n* = 3). The analysis revealed significant differences among mixtures at both ages (28 d: *F*(8, 18) = 5.66, *p* = 0.001; 56 d: *F*(8, 18) = 7.18, *p* < 0.001) (Table 7).

**Table 7:** Tukey HSD homogeneous subsets for Group III mixtures.

Age	Mix	*N*	Subset for alpha = 0.05		
			A	B	C
28 days	Mix 15 (6% BN + 5% SF)	3	38.27		
	Mix 18 (6% BN + 10% SF)	3	41.08	41.08	
	Mix 13 (2% BN + 5% SF)	3	41.13	41.13	
	Mix 14 (4% BN + 5% SF)	3		43.24	43.24
	Mix 16 (2% BN + 10% SF)	3		44.34	44.34
	Mix 21 (6% BN + 15% SF)	3		45.36	45.36
	Mix 17 (4% BN + 10% SF)	3		45.79	45.79
	Mix 19 (2% BN + 15% SF)	3		47.26	47.26
	**Mix 20 (4% BN + 15% SF)**	3			**49.04**
	**Sig.**		**0.124**	**0.112**	**0.157**
56 days	Mix 15 (6% BN + 5% SF)	3	42.04		
	Mix 18 (6% BN + 10% SF)	3	46.29	46.29	
	Mix 13 (2% BN + 5% SF)	3	46.94	46.94	
	Mix 14 (4% BN + 5% SF)	3		47.43	47.43
	Mix 21 (6% BN + 15% SF)	3		49.07	49.07
	Mix 16 (2% BN + 10% SF)	3		50.80	50.80
	Mix 19 (2% BN + 15% SF)	3		52.51	52.51
	Mix 17 (4% BN + 10% SF)	3		52.91	52.91
	**Mix 20 (4% BN + 15% SF)**	3			**54.27**
	**Sig.**		**0.058**	**0.085**	**0.070**

**Fig. 15:**
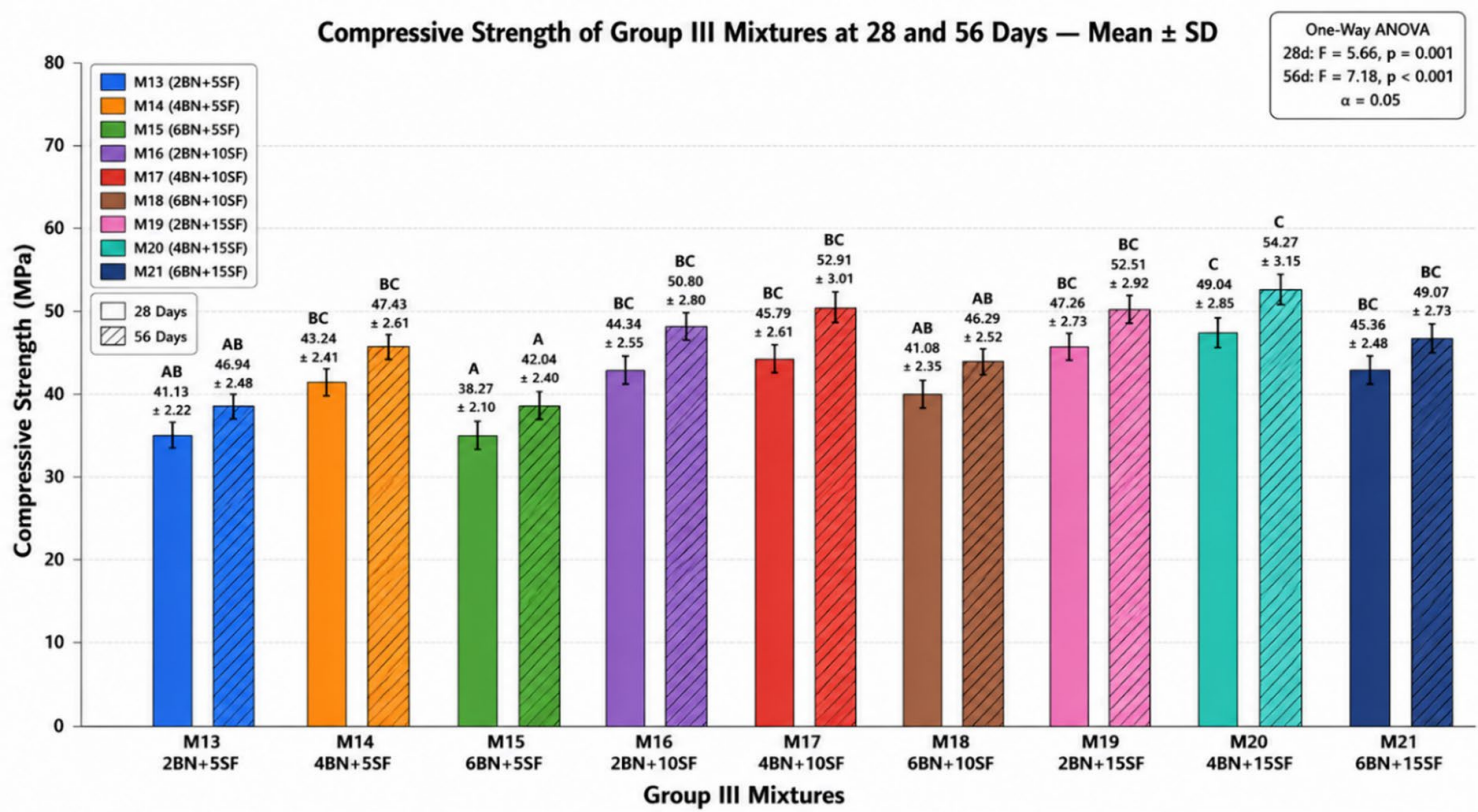
Compressive strength of Group III mixtures at 28 and 56 days (Mean ± SD).

The Tukey HSD analysis indicated that Mix 20 (4% bentonite + 15% silica fume) achieved the highest compressive strength among all Group III mixtures at both curing ages, differing significantly from the lower-performing mixtures and confirming its superior performance (Fig. 15).

### Tensile and flexural strength

The 28-day splitting tensile and flexural strengths for all 22 mixtures are summarized in Table 8, together with the corresponding 56-day compressive strengths for comparative reference. The control mixture exhibited tensile and flexural strengths of 3.70 MPa and 4.30 MPa, respectively.

Among all mixtures, Mix 20 (4% bentonite + 15% silica fume) achieved the highest tensile strength of 5.00 MPa, representing a 35.1% improvement over the control. For flexural strength, the top performers were Mix 20 (6.87 MPa, 59.8% improvement), Mix 17 (4% bentonite + 10% silica fume) (6.40 MPa, 48.8%), and Mix 19 (2% bentonite + 15% silica fume) (6.30 MPa, 46.5%).

**Table 8:** Splitting tensile strength and flexural strength of selected concrete mixtures at 28 days.

Mixture	Tensile (MPa)	Flexural (MPa)	Comp. 56d (MPa)	Group
Control	3.70	4.30	41.80	OPC
Mix 1	3.85	4.65	44.53	I
Mix 12	2.85	3.05	31.88	II
Mix 17	4.60	6.40	52.91	III
Mix 20	5.00	6.87	54.27	III

**Tensile Strength (28 days)**: A one-way between-groups ANOVA comparing 28-day tensile strength across the four concrete groups (Control: 100% OPC, *n* = 1; Group I: bentonite only, *n* = 3; Group II: bentonite + fly ash, *n* = 9; Group III: bentonite + silica fume, *n* = 9) revealed a statistically significant difference (*F* = 25.79, *p* < 0.001). Levene’s test confirmed homogeneity of variances (*p* = 0.891). The effect size was large (eta-squared = 0.81), indicating that group membership explained approximately 81% of the variance in tensile strength (Tables 9, 10).

**Table 9:** Tukey HSD homogeneous subsets for 28-day tensile strength (between groups).

Age	Group	*N*	Subset for alpha = 0.05	
			A	B
28 days	Group II (Bentonite + Fly Ash)	9	3.32	
	Group I (Bentonite only)	3	3.76	
	Group III (Bentonite + Silica Fume)	9		4.50
	Sig.		0.054	1.000

**Table 10:** Tukey HSD pairwise comparisons for 28-day tensile strength (between groups).

Comparison	Mean difference (MPa)	Std. error	*p*-value	95% CI Lower	95% CI Upper	Sig
Group I vs. Group II	0.44	0.19	0.082	-0.05	0.93	ns
Group I vs. Group III	-0.74	0.19	0.003	-1.23	-0.25	**
Group II vs. Group III	-1.18	0.14	< 0.001	-1.53	-0.84	***

**Fig. 16:**
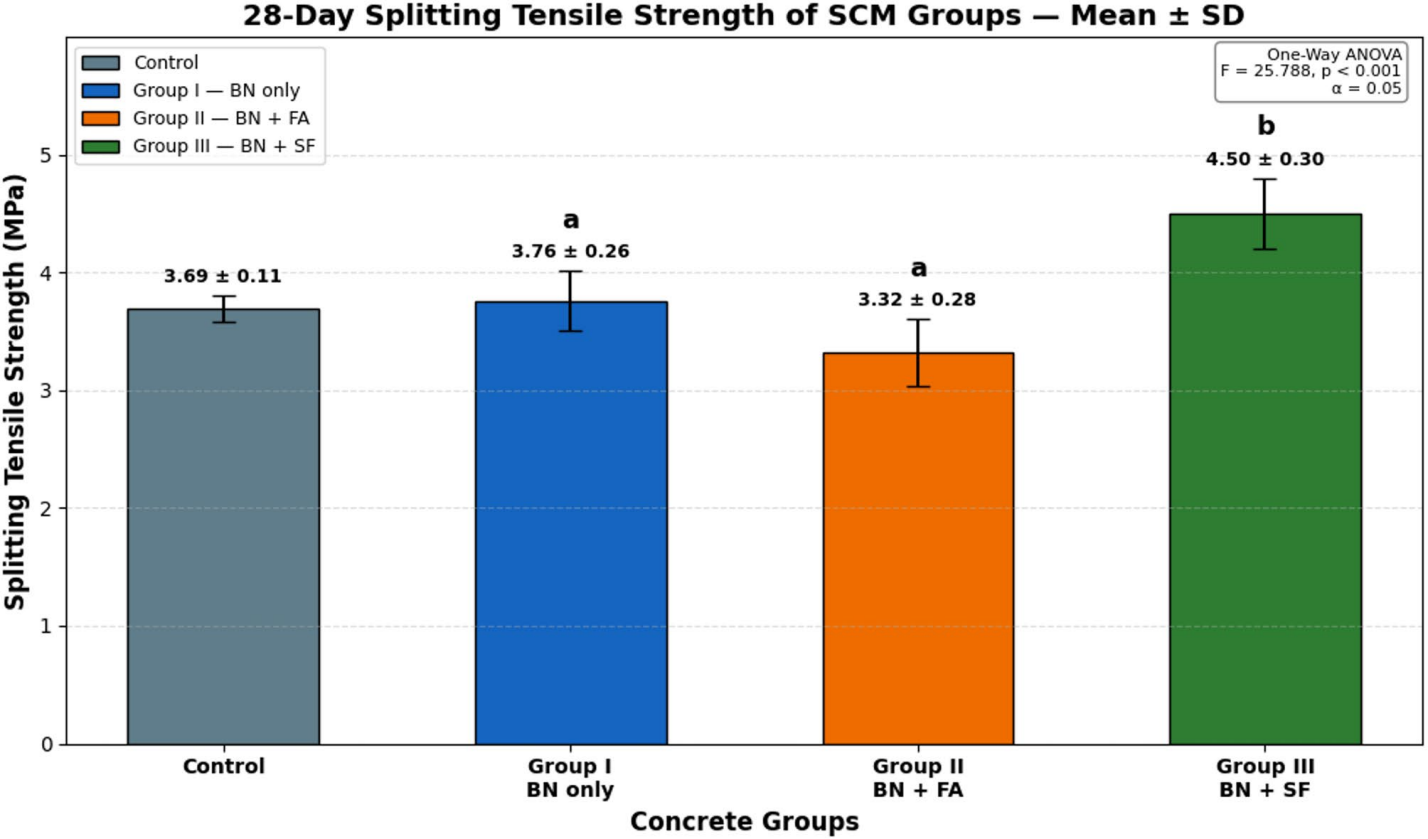
The 28-day splitting tensile strength of SCM groups (Mean ± SD).

Post-hoc comparisons using Tukey HSD showed that Group III (bentonite + silica fume) achieved significantly higher tensile strength than both Group II (MD = 1.18 MPa, *p* < 0.001) and Group I (MD = 0.74 MPa, *p* = 0.003). No significant difference was observed between Group I and Group II (MD = 0.44 MPa, *p* = 0.082). The homogeneous subsets analysis placed Group III in the highest-performing subset (4.50 MPa), Group II in the lowest-performing subset (3.32 MPa), and Group I in an intermediate position (3.76 MPa), which was statistically similar to Group II (*p* = 0.054). These findings demonstrate that the synergistic combination of bentonite and silica fume significantly enhanced tensile strength compared with both the bentonite-only and fly-ash-containing systems (Fig. 16).

#### Flexural strength (28 days)

A one-way between-groups ANOVA comparing 28-day flexural strength across the same four groups also yielded a statistically significant result (*F* = 25.79, *p* < 0.001), with Levene’s test confirming homogeneity of variances (*p* = 0.814). The effect size was large (eta-squared = 0.82), indicating that approximately 82% of the variance in flexural strength was attributable to group membership (Tables 11, 12).

**Table 11:** Tukey HSD homogeneous subsets for 28-day flexural strength (between groups).

Age	Group	*N*	Subset for alpha = 0.05		
			A	B	C
28 days	Group II (Bentonite + Fly Ash)	9	3.68		
	Group I (Bentonite only)	3		4.46	
	Group III (Bentonite + Silica Fume)	9			5.95
	**Sig.**		**1.000**	**1.000**	**1.000**

**Table 12:** Tukey HSD pairwise comparisons for 28-day flexural strength (between groups).

Comparison	Mean difference (MPa)	Std. error	*p*-value	95% CI Lower	95% CI Upper	Sig
Group I vs. Group II	0.78	0.36	0.103	-0.14	1.70	ns
Group I vs. Group III	-1.49	0.36	0.002	-2.40	-0.57	**
Group II vs. Group III	-2.27	0.25	< 0.001	-2.92	-1.62	***

**Fig. 17:**
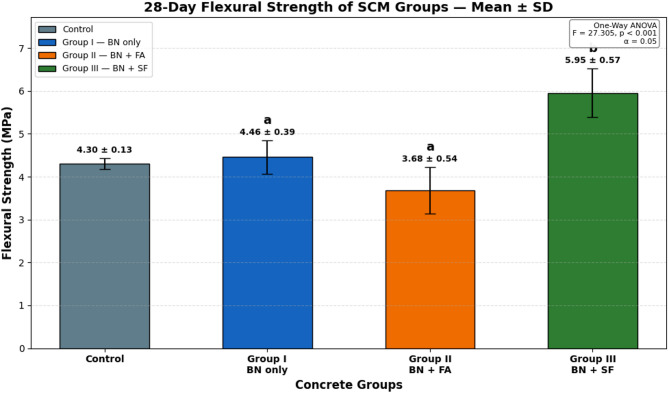
The 28-Day flexural strength of SCM groups — Mean ± SD.

Post-hoc Tukey HSD comparisons revealed the same pattern of group differences for flexural strength as was observed for tensile strength. Group III significantly outperformed both Group II (MD = 2.27 MPa, *p* < 0.001) and Group I (MD = 1.49 MPa, *p* = 0.002). In comparison, the difference between Group I and Group II was not statistically significant (MD = 0.78 MPa, *p* = 0.103). The homogeneous subsets analysis placed each group in a separate subset: Group III in the highest tier (5.95 MPa), Group I in the intermediate tier (4.46 MPa), and Group II in the lowest tier (3.68 MPa). These results confirm that the bentonite–silica fume system provides substantial gains in flexural strength relative to both bentonite-only and fly ash-modified mixtures (Fig. 17).

#### Coefficient of variation (CV%) analysis

The coefficient of variation (CV%) was calculated to assess the relative variability of compressive strength measurements across the four experimental groups. CV% values below 15% are generally considered acceptable in concrete research and indicate satisfactory repeatability (Table 13).

**Table 13:** Coefficient of Variation (CV%) for 28-day and 56-day compressive strength (between groups).

Group	*N*	28-day Mean (MPa)	28-day SD (MPa)	CV% (28d)	56-day Mean (MPa)	56-day SD (MPa)	CV% (56d)
Control	1	36.76	1.08	2.94%	41.80	1.15	2.75%
Group I (Bentonite only)	3	36.80	2.08	5.65%	41.89	4.91	11.72%
Group II (Bentonite + Fly Ash)	9	32.59	3.47	10.65%	37.12	3.80	10.24%
Group III (Bentonite + Silica Fume)	9	43.95	3.38	7.69%	49.14	3.89	7.92%

All groups exhibited CV% values below 12%, confirming good repeatability and low experimental variability. Group I showed relatively higher variability at 56 days (CV% = 11.72%), attributable to its smaller sample size (*n* = 3), whereas Group III demonstrated consistently low variability at both ages (CV% < 8%).

### Seawater (sulfate) resistance

Durability performance under aggressive marine conditions was assessed by comparing the compressive strengths of specimens cured in tap water versus seawater. Most mixtures exhibited similar compressive strengths under both curing regimes, indicating satisfactory sulfate resistance.

**Fig. 18:**
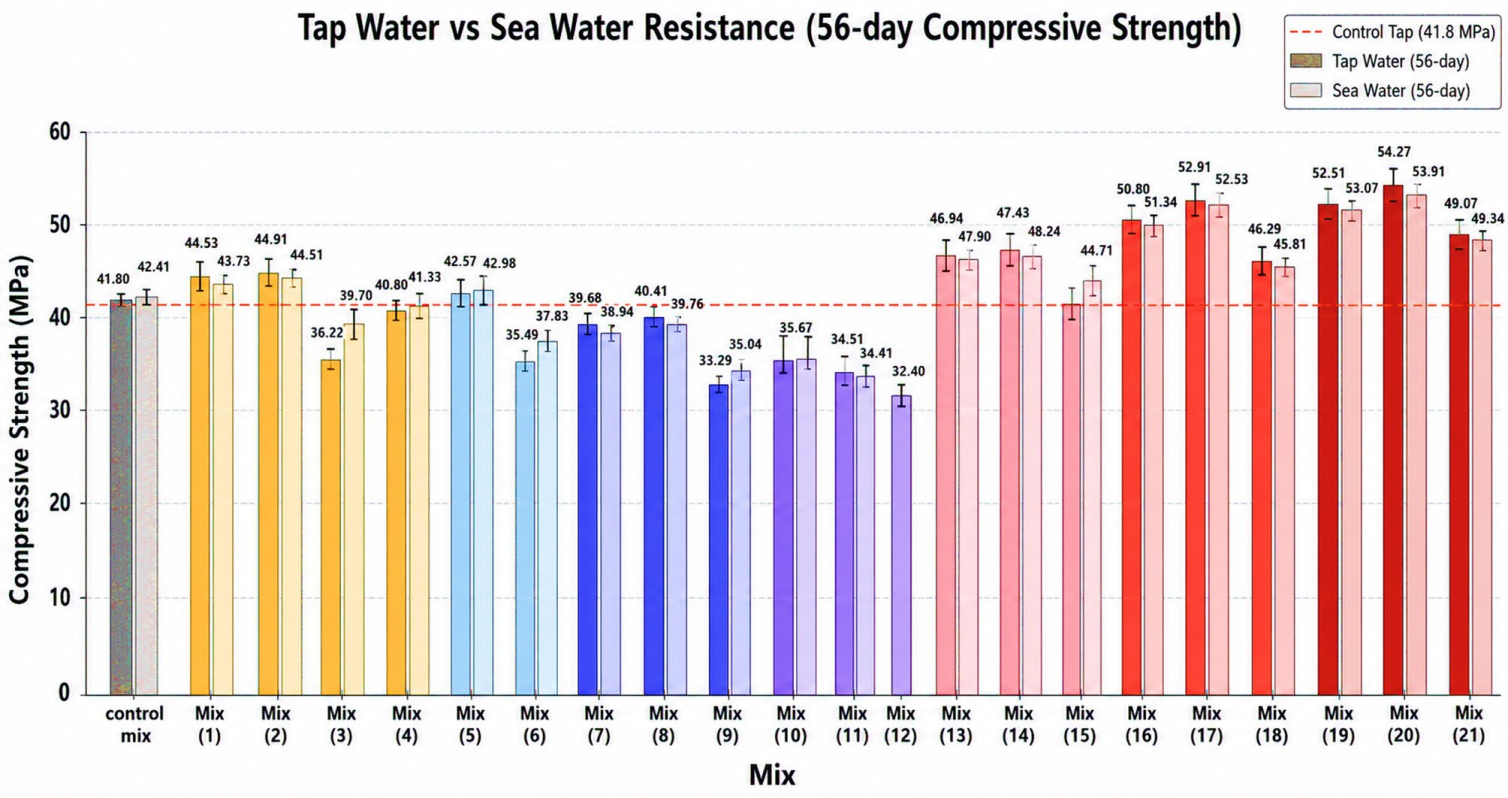
Compressive strength of the mixes after 56 days cured in tap water and seawater.

Mix 20 demonstrated the highest resistance to seawater exposure, achieving a compressive strength of 53.9 MPa at 56 days after seawater curing, compared with 54.3 MPa for the tap-water-cured counterpart—an insignificant 0.7% loss in strength (Fig. 18).

### Rapid chloride permeability test (RCPT)

Chloride ion penetration resistance was evaluated using the Rapid Chloride Permeability Test (RCPT) at 56 days in accordance with ASTM C1202-22. SCM incorporation markedly reduced chloride permeability, with the greatest improvements observed in ternary mixtures containing bentonite and silica fume. Mix 19 achieved the lowest recorded value (250 Coulombs) compared with the control (2118 Coulombs), corresponding to a classification of “very low” chloride permeability per ASTM criteria (Table 14).

**Table 14:** Representative RCPT results of selected concrete mixtures at 56 days (ASTM C1202-22).

Group	Mix	RCPT (Coulombs)	ASTM classification
OPC	Control	2118	Moderate
I	Mix 1	1946	Low
II	Mix 12	525	Very Low
III	Mix 16	435	Very Low
III	Mix 19	250	Very Low

## Results: environmental impact (LCA)

### Global warming potential (GWP)

**Fig. 19:**
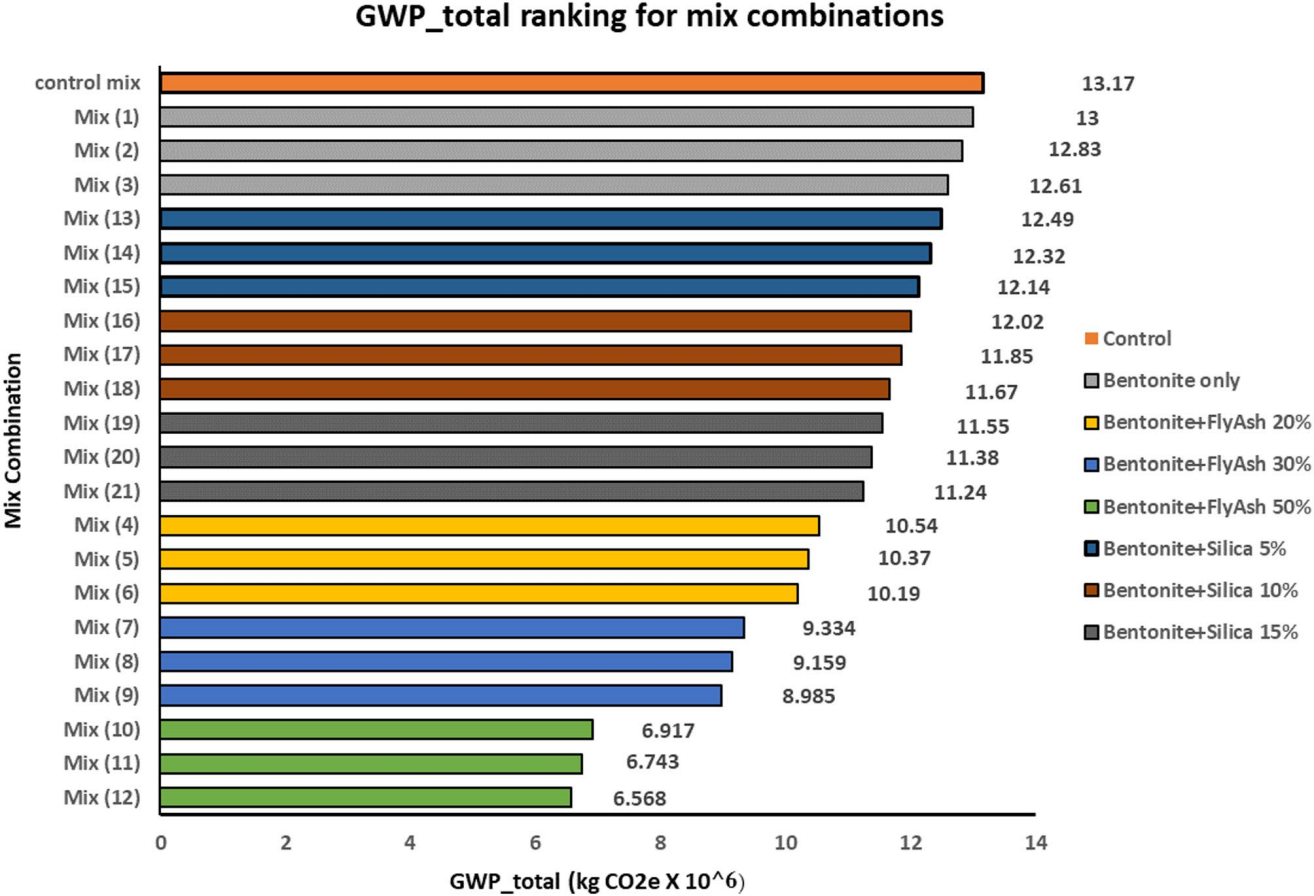
Global warming potential (GWP) ranking of all investigated concrete mixtures based on life cycle assessment results.

The global warming potential (GWP) of each concrete mixture was evaluated using lifecycle assessment (LCA). The GWP results for all mixtures are compared in Fig. 19. These values represent the total lifecycle greenhouse gas emissions attributable to each concrete mixture.

The control mixture had the highest GWP of approximately 13.17 × 10^^6^ kg CO₂-eq, reflecting its elevated cement content. The incorporation of supplementary cementitious materials (SCMs) produced substantial reductions in the environmental impact of the evaluated mixtures.

### The ODP anomaly in silica fume ternary mixtures

The ozone depletion potential (ODP) results revealed a clear divergence in environmental behavior between the two SCM groups. Groups I and II (bentonite only and bentonite + fly ash) showed a uniform reduction in ODP, with decreases ranging from 0.19% to 23.64% relative to the control, reflecting the environmental benefits of partial cement replacement with SCMs.

**Fig. 20:**
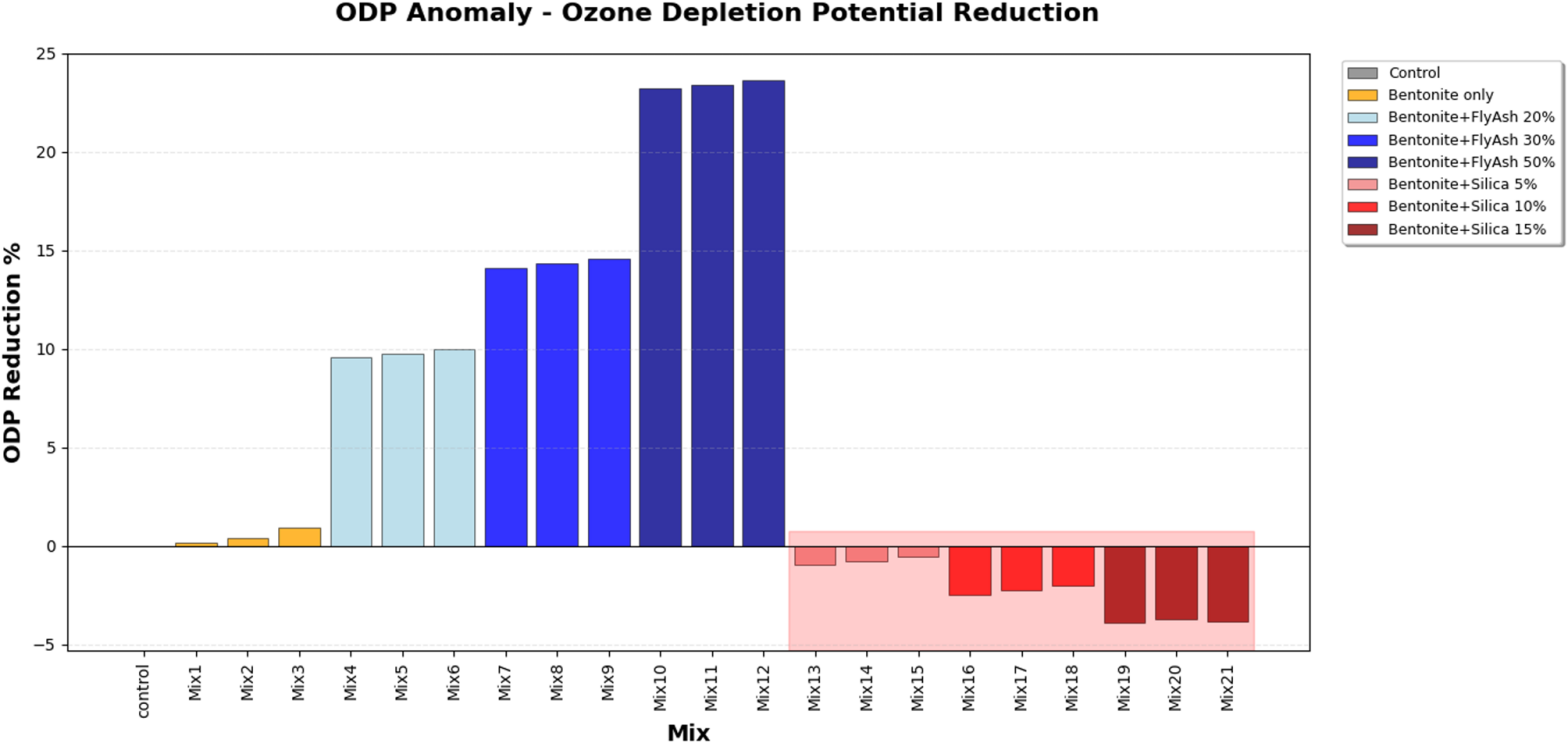
ODP (kg CFC-11e) for all 22 mixtures. The red zone (mixes 13–21) shows an ODP anomaly.

In contrast, Group III mixtures (all containing silica fume in addition to bentonite) exhibited a paradoxical increase in ODP relative to the control, ranging from 0.56% (Mix 16) to 3.90% (Mix 21). The magnitude of this increase appeared to intensify with higher silica fume contents. However, this trend should be interpreted with caution, as the environmental impacts attributed to industrial byproducts can be highly sensitive to allocation assumptions and background database methodologies employed in LCA modeling (Fig. 20).

### Multi-category environmental heatmap analysis

**Fig. 21:**
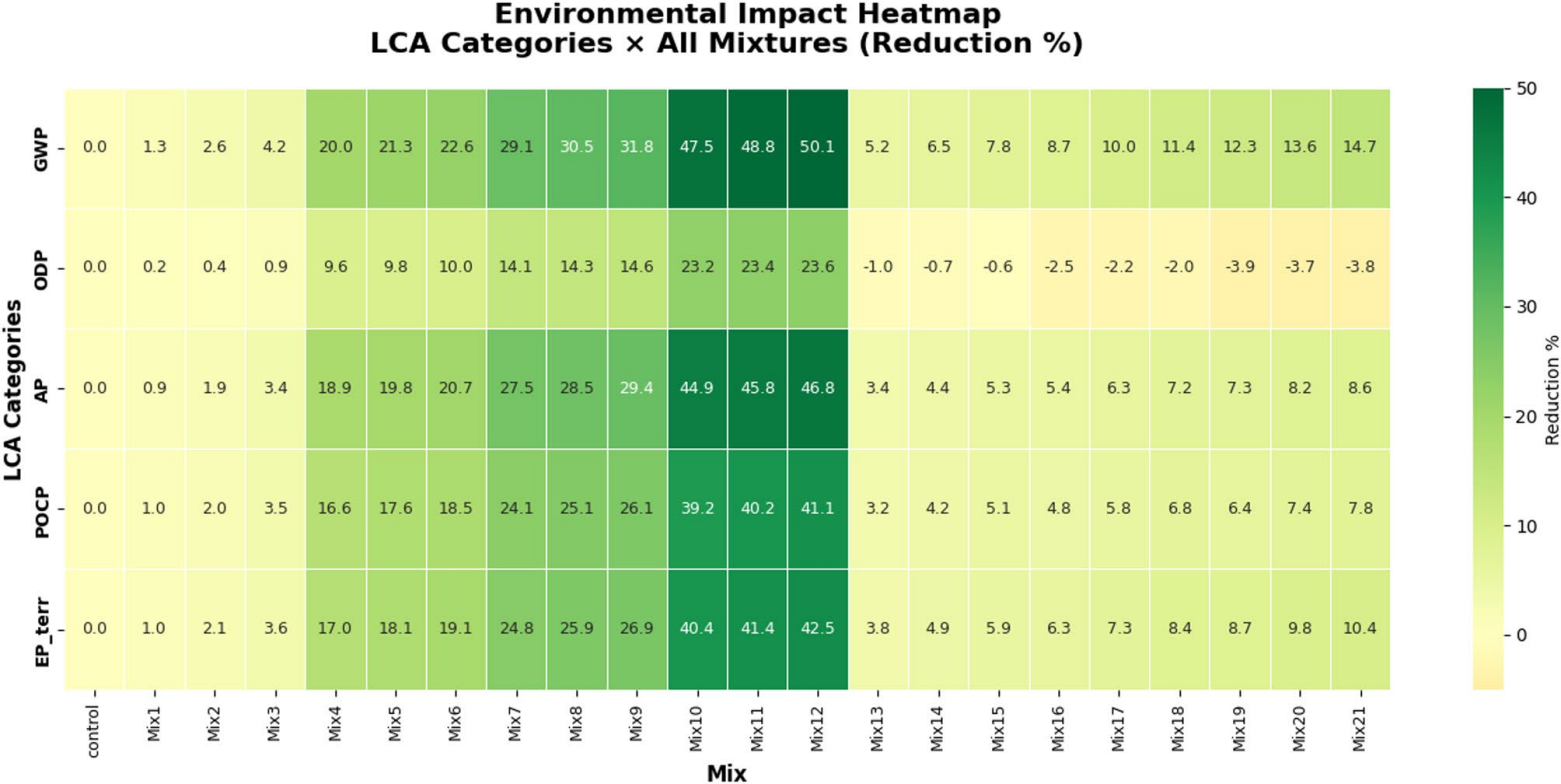
Heatmap of min–max normalized environmental reduction percentages across GWP, ODP, AP, EP, and POCP.

For Groups I and II (bentonite only and bentonite + fly ash), all five impact categories decreased proportionally with the level of OPC replacement. Mix 12 (50% fly ash) achieved the greatest reductions across all impact categories, with savings of 50.1% for GWP, 46.8% for AP, 41.1% for POCP, and 42.5% for EP_terr_ (Fig. 21).

### Life cycle stage contribution

**Fig. 22:**
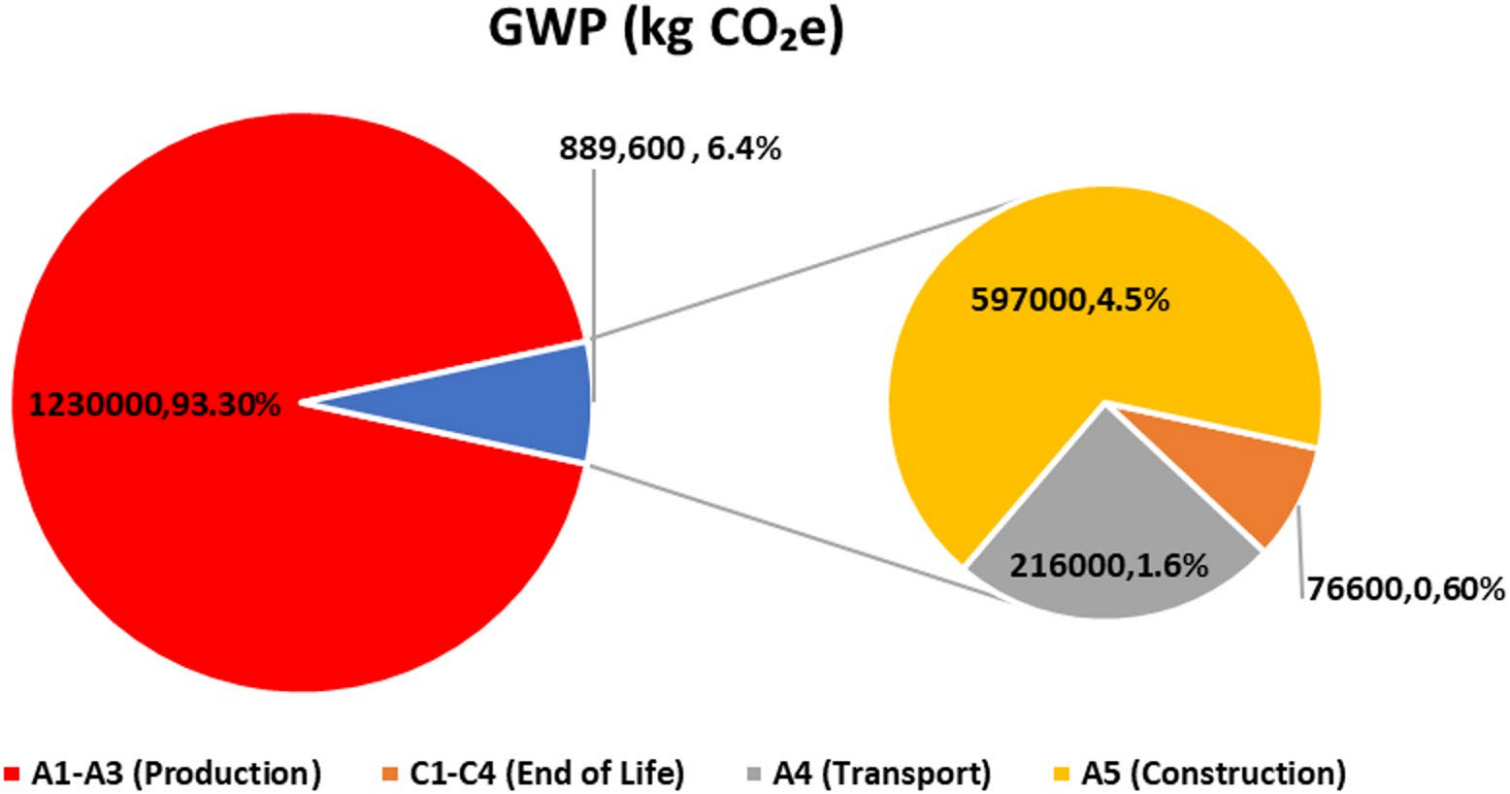
Distribution of global warming potential across life cycle stages (A1–A3, A4, A5, and C1–C4) for all investigated concrete mixtures.

The contribution of individual life cycle stages to total emissions is illustrated in Fig. 22. Modules A1–A3 (raw material supply, transport, and manufacturing) accounted for the majority of the GWP for all investigated mixtures, constituting the principal source of environmental impact across the life cycle.

### GWP–EP correlation and cement as master variable

**Fig. 23:**
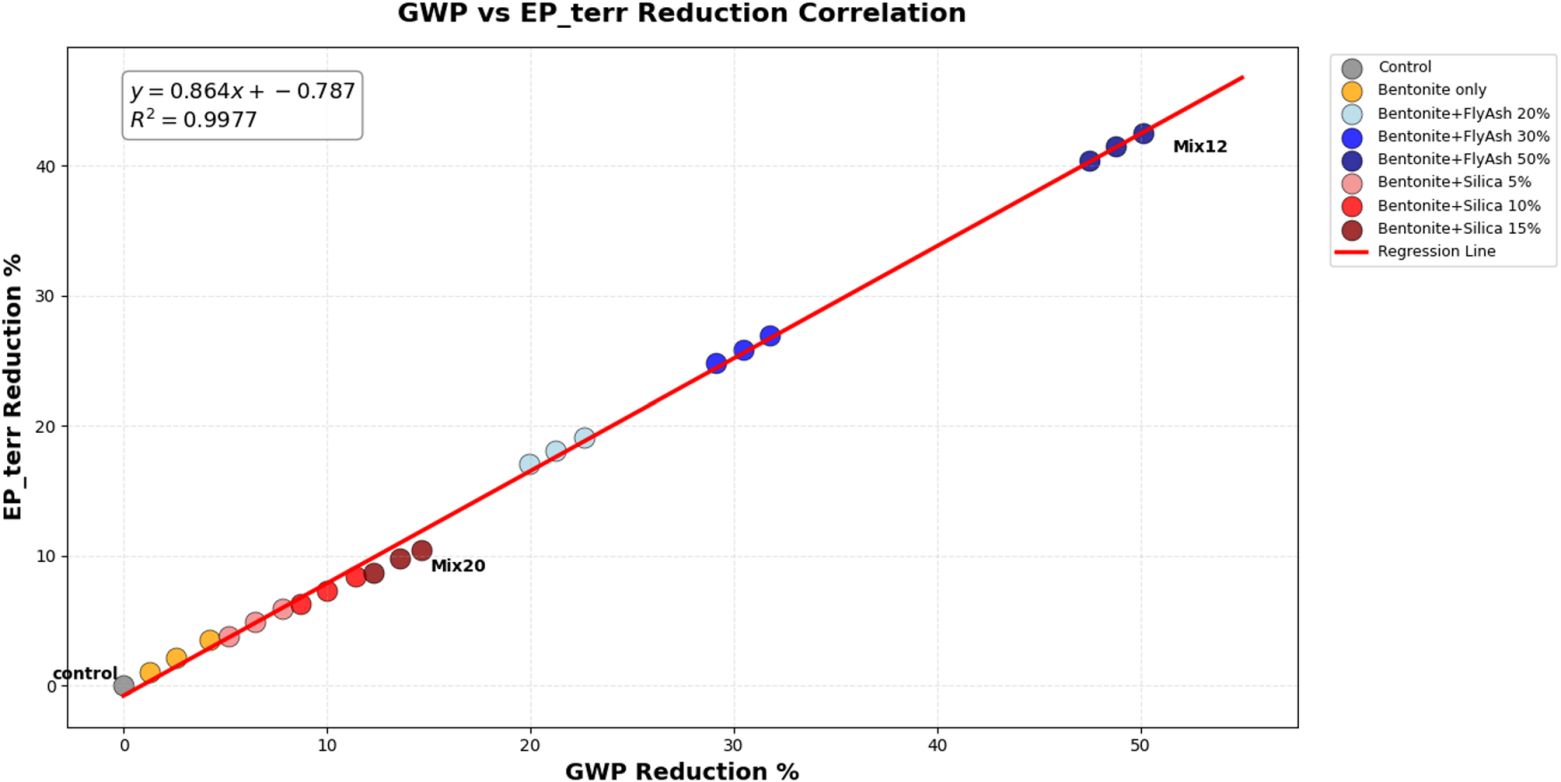
Pearson correlation between GWP and EPterr for all 22 mixtures (*r* = 0.999, *p* < 0.001).

The Pearson correlation between GWP and EP_terr_ for the 22 mixtures is presented in Fig. 23. A near-perfect positive correlation was observed (*r* = 0.999, *p* < 0.001), indicating that these two impact categories are almost perfectly linearly related across the evaluated mixtures.

### Environmental impact categories—overall summary

**Fig. 24:**
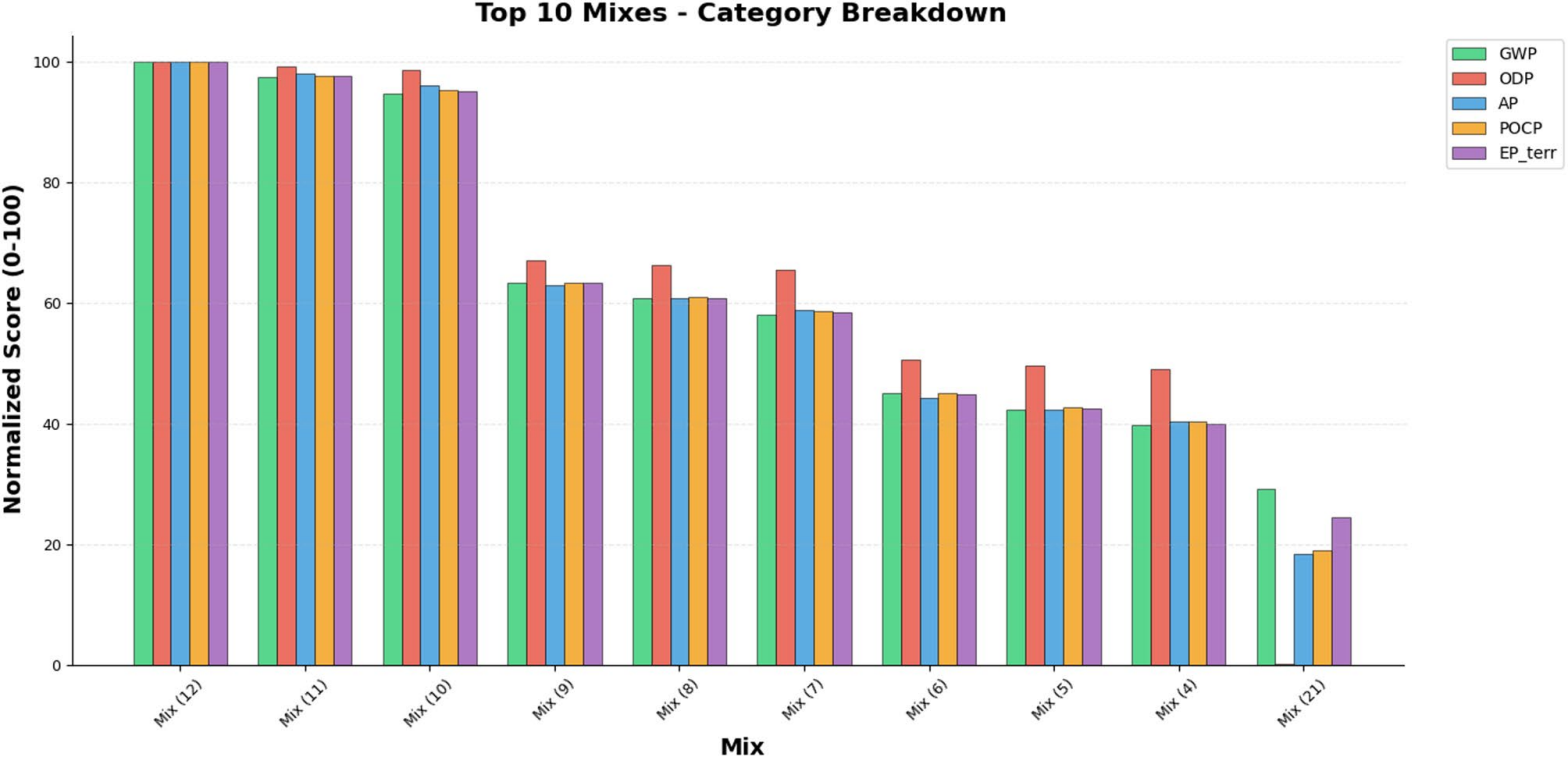
Normalized environmental impact scores for the top ten concrete mixtures across multiple impact categories.

The environmental performance of the top ten mixtures across all impact categories is shown in Fig. 24 as normalized environmental scores. The scores are based on the following parameters: GWP, ODP, AP, POCP, and EP.

## Integrated environmental–mechanical analysis

### Pareto Frontier analysis

**Fig. 25:**
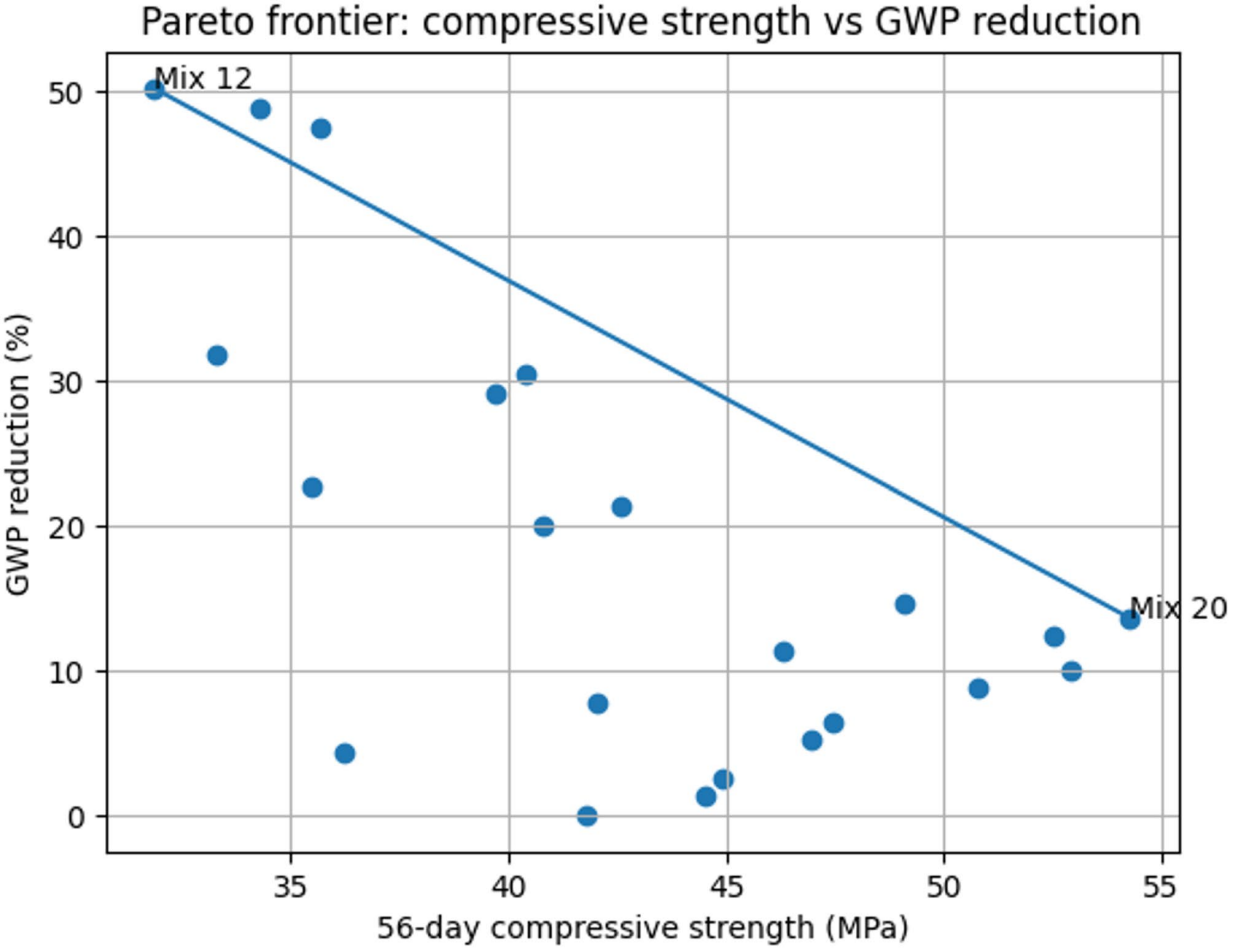
Pareto frontier analysis of all 22 concrete mixtures in the 56-day compressive strength versus GWP reduction space.

All 22 mixtures were positioned in a bimodal performance space of 56-day compressive strength (mechanical performance) versus GWP mitigation (environmental performance). Pareto frontier analysis was used to identify the set of non-dominated solutions (Fig. 25).

Two non-dominated solutions were identified, representing the extremes of the performance spectrum:

- *Mixture 12 (50% fly ash)* 56-day strength of 31.88 MPa and GWP reduction of 50.13% (environmental Pareto optimum).
- *Mixture 20 (4% bentonite + 15% silica fume)* 56-day compressive strength of 54.27 MPa at a GWP reduction of 13.59% (structural Pareto optimum).

### Policy regression: SCM replacement→GWP reduction

A linear regression analysis was conducted to determine how well the total cement replacement percentage predicted GWP reduction across the 21 SCM mixtures (excluding the control). The resulting equation was:

$$\:\mathrm{GWP\:Reduction\:(\%)\:=\:}0.9417\times\:\mathrm{Cement\:Replacement\:(\%)\:}-2.2126$$

$$\:\:\left[{R}^{2}=0.9938,\:\:p<0.001\right]$$

**Fig. 26:**
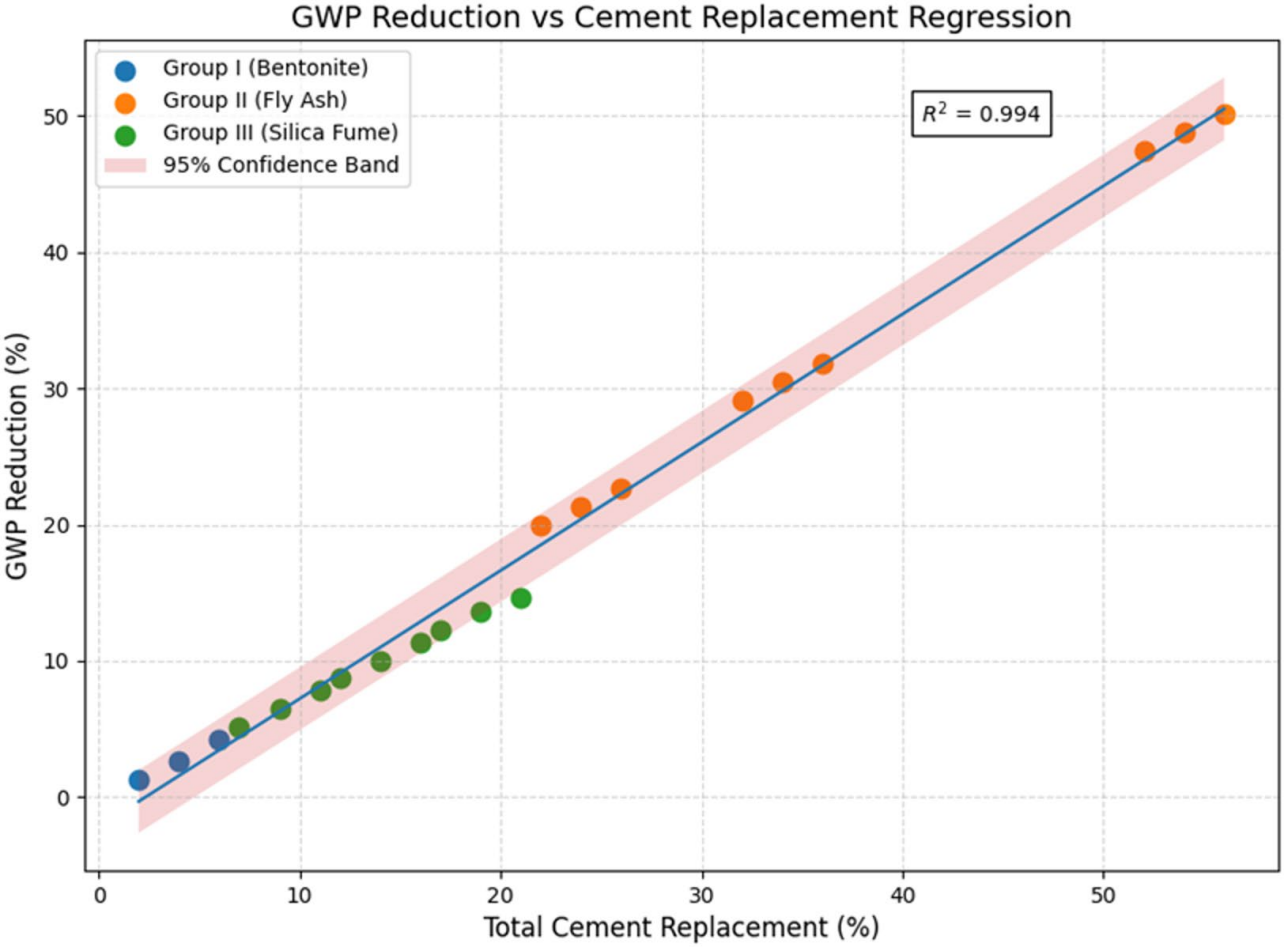
Linear regression of GWP reduction versus cement replacement for all SCM mixtures (R² = 0.994, *p* < 0.001).

In engineering terms, the slope (0.9417) indicates that a 1% increase in cement replacement results in an approximate 0.94% reduction in GWP. It confirms the dominant contribution of clinker production to the embodied carbon of concrete mixtures. The regression model was developed using fixed Egyptian regional boundaries and standardized transport assumptions. Longer transport distances or alternative material sourcing would increase transportation-related environmental burdens and affect overall GWP values (Fig. 26).

### Practical mix selection framework

Based on the integrated analysis, an application-specific mix selection framework was developed to guide engineers and decision-makers in selecting the most appropriate concrete mixture for different project requirements. Table 15 summarizes the recommended mixtures based on various application priorities.

**Table 15:** Application-specific mix selection framework based on integrated mechanical and environmental performance.

Application priority	Mix	Key metrics	Rationale
Maximum structural	Mix 20	54.3 MPa, 6.87 MPa flexural	Pareto-optimal for strength
Marine exposure	Mix 19	52.5 MPa, 53.1 MPa (seawater)	Superior seawater durability
Balanced performance	Mix 17	52.9 MPa, 10.0% GWP reduction	Best composite performance
Maximum environmental	Mix 12	31.9 MPa, 50.1% GWP reduction	Pareto-optimal for GWP
ODP-sensitive	Mix 11–12	ODP reduction: 23.4–23.6%	No ODP anomaly

## Discussion

### Mechanical performance

The 56-day compressive strengths of all mixtures satisfied common structural concrete requirements (C25/30 or higher), indicating that most mixtures are suitable for structural applications. Group III mixtures achieved the highest compressive strengths due to the ultra-fine particles and amorphous silica content of silica fume, which improved particle packing, refined the microstructure, and strengthened the interfacial transition zone (ITZ) through additional calcium–silicate–hydrate (C–S–H) formation^30,34^. Specifically, this superior mechanical performance is driven by two continuous and overlapping mechanisms: the physical micro-filler effect and the chemical pozzolanic reaction. Physically, the sub-micron particles of silica fume (SF) occupy the microscopic interstitial voids between larger cement grains, establishing a highly homogenous particle-size distribution. This early physical densification acts as a spatial catalyst, providing extensive nucleation sites that accelerate early-age hydration kinetics^35^. Chemically, the highly reactive amorphous silica in SF rapidly consumes the weak, crystalline calcium hydroxide (Ca(OH)₂) byproducts liberated during Portland cement hydration, transforming them into secondary, high-density C–S–H gels. The high tensile and flexural strengths observed in Group III further confirm the beneficial role of silica fume in ITZ densification, particularly under tensile loading conditions. This interpretation is supported by the strong Pearson correlation (*r* = 0.97) between splitting tensile strength and 56-day compressive strength. Additionally, silica fume mixtures demonstrated superior durability due to their dense pore structure and reduced chloride penetration, making them suitable for aggressive marine environments^30,36^. This pore refinement and particle packing optimization drastically reduce capillary pore connectivity, leading to reduced permeability and superior resistance to aggressive ions under marine exposure conditions^37^.

Mix 20 consistently occupied the outermost position across all performance indicators, confirming it as the optimum mixture. This behavior is attributed to the synergistic interaction between bentonite and silica fume, which enhanced particle packing, pore refinement, and ITZ densification. The 59.8% increase in flexural strength for Mix 20 exceeded values commonly reported for binary SCM systems^38^.

The improved performance of the optimized mixtures can also be explained using Particle Packing Theory and the Modified Andreasen-Andersen (A&A) model. The combination of bentonite with fly ash or silica fume resulted in a more continuous particle-size distribution, reduced internal voids, and increased packing density within the cementitious matrix. This filler effect reduced initial porosity and synergistically enhanced mechanical performance through pozzolanic reactions^39,40^.

Group I showed a narrow interquartile range, indicating stable compressive strength behavior. This stability is associated with bentonite’s fine particle size, which provides nucleation sites and enhances matrix densification^26,41^. However, the high loss on ignition (LOI) and swelling behavior of bentonite under wet–dry cycles may raise concerns about long-term shrinkage and dimensional stability^42^. These effects are expected to be limited because bentonite replacement levels remained low (2–6%). Nevertheless, autogenous and drying shrinkage have not been experimentally evaluated and should be investigated in future studies. The favorable performance of bentonite-containing mixtures is consistent with the findings of Kim et al.^15^, who reported that thermally activated bentonite can exhibit significant pozzolanic activity and contribute to strength development through the formation of additional cementitious reaction products.

Group II mixtures showed larger variability, reflecting the dose-dependent behavior of fly ash. The 50% fly ash mixture (Mix 12) recorded the lowest compressive strength (31.9 MPa). This pronounced strength drop-off at higher replacement levels is fundamentally governed by the dilution effect and the inherently sluggish kinetics of the fly ash pozzolanic reaction, which severely delay early and intermediate strength evolution^43^. At elevated replacement rates, the initial volume of Ordinary Portland Cement (OPC) is significantly reduced. Because fly ash relies directly on the calcium hydroxide (Ca(OH)₂) liberated during OPC hydration to trigger its secondary reactions, this reduction creates a chemical deficit that hinders early hydration dynamics and slows down strength development^43^. Several studies have reported that fly ash replacement levels exceeding approximately 30% may result in diminishing mechanical performance due to dilution effects and delayed pozzolanic activity^44^. Past this optimum limit, a substantial portion of the fly ash particles may remain partially unreacted within the matrix up to 56 days. Consequently, the contribution of fly ash to secondary C-S-H formation becomes insufficient to compensate for the reduction in OPC content, resulting in the observed decline in compressive strength. Continued pozzolanic reactions may contribute to long-term strength development. This limitation can be mitigated by using finer fly ash particles or elevated-temperature curing^45–47^.

Similar trends have been reported for other SCM systems. Rathore and Raheem^6^ observed that rice husk ash improved compressive, tensile, and flexural strengths up to an optimum replacement level of approximately 20%, beyond which performance declined due to reduced cement content and slower reaction kinetics. This behavior supports the existence of an optimum SCM replacement threshold that balances sustainability and mechanical performance.

Despite lower early-age strength, fly ash mixtures demonstrated good resistance under marine exposure conditions while significantly reducing environmental impacts by lowering cement consumption^36,48^. Comparable improvements in durability have been reported for concrete incorporating rice husk ash, metakaolin, and manufactured sand, where enhanced microstructural densification reduced permeability and improved resistance to sulfate and acid attack^5,7,12^.

.

This favorable seawater resistance behavior can be attributed to several coupled physical and chemical mechanisms governed by the ternary blend. Physically, the synergistic effect of bentonite, fly ash, and silica fume contributes to pore refinement, reducing the connectivity of capillary pores and the tortuosity of the matrix, effectively limiting the ingress of aggressive chloride and sulfate ions. In addition, the enhanced pozzolanic reactivity accelerates the formation of supplementary C–S–H and C–A–S–H gels, contributing to a denser cementitious microstructure with lower permeability. Chemically, both fly ash and bentonite enhance chloride binding by forming Friedel’s salt, which immobilizes free chloride ions and reduces their diffusion coefficient in concrete. Simultaneously, the intensive consumption of calcium hydroxide (Ca(OH)₂) during the prolonged pozzolanic reactions drastically decreases the availability of vulnerable phases susceptible to sulfate attack (such as gypsum and secondary ettringite formation), thereby substantially improving durability under marine exposure conditions^49–52^.

From an economic perspective, bentonite is a relatively low-cost, locally available supplementary cementitious material^53^. Although silica fume is generally more expensive, its ability to improve durability and resistance to aggressive exposure conditions may justify the initial cost through reduced long-term maintenance and rehabilitation requirements^54^. Similar conclusions have been reported for concrete incorporating recycled steel slag and ground granulated blast furnace slag, where improved durability performance offset part of the initial material costs through enhanced service life and reduced maintenance requirements^8^.

### Environmental impact (LCA)

Cement production is the principal contributor to concrete’s greenhouse gas emissions, driven by energy-intensive clinker production and limestone calcination. Accordingly, the fly ash mixtures showed substantial reductions in GWP because fly ash is a coal combustion byproduct that requires no additional manufacturing. Even small reductions in cement content can significantly reduce total GWP, as cement accounts for nearly 90% of concrete-related emissions^55^.

The LCA results showed a moderate increase in ODP values in mixtures containing silica fume. This trend became more pronounced as silica fume replacement increased from 5% to 10%. Within the adopted LCA framework, these results are strongly influenced by the economic allocation procedures and background database assumptions used in Ecoinvent/One Click LCA. In global inventory datasets, a portion of the environmental burden of metallurgical production is allocated to industrial byproducts, such as silica fume, based on their market value relative to primary alloys.

Similar observations have been reported in previous studies. Timm et al.^56^ demonstrated that the environmental impacts assigned to silica fume vary depending on the selected allocation method and impact distribution models used in LCA databases. Recent literature also indicates that some LCA databases may overestimate ozone depletion impacts because the phase-out of ozone-depleting substances under the Montreal Protocol is not fully reflected in current characterization models^57^. Therefore, the observed ODP increase reflects the adopted database configuration and allocation methodology rather than the intrinsic environmental performance of silica fume itself.

A cradle-to-grave system boundary with a 60-year service life (excluding B1–B7 stages) was selected to provide a more comprehensive environmental assessment than a cradle-to-gate approach. A cradle-to-gate assessment includes only the material production and manufacturing stages (A1–A3), whereas the adopted framework also incorporates end-of-life stages (C1–C4). This approach is particularly important for marine infrastructure applications, where durable ternary mixtures may provide long-term environmental benefits over the full service life.

### Integrated environmental–mechanical analysis

Pareto frontier optimization was used to support multi-criteria decision-making under conflicting design requirements. Sustainable concrete design requires balancing mechanical performance with environmental impact. This approach identifies balanced optimal solutions and supports practical engineering decisions.

The non-dominated status of Mix 12 and Mix 20 confirms the existence of two distinct optimization routes. Mix 12 (environmental Pareto optimum) achieves maximum GWP reduction (50.1%) but with low compressive strength (31.88 MPa), limiting its use to non-structural applications. Mix 20 (structural Pareto optimum) achieves maximum compressive strength (54.27 MPa) via synergistic bentonite–silica fume effects, with a modest but significant 13.6% GWP reduction.

The experimental dataset can also support constrained optimization for selecting low-carbon concrete mixtures. When a minimum compressive strength requirement is defined (e.g., 35 MPa), the optimization objective is to minimize GWP while satisfying structural performance requirements:

$$\:\mathrm{Minimize\:GWP}=f\left(\mathrm{BN},\:\mathrm{SF},\:\mathrm{FA}\right)\:\mathrm{subject\:to}\:{f}_{cu}\left(\mathrm{BN},\:\mathrm{SF},\:\mathrm{FA}\right)\ge\:35\:\mathrm{MPa}$$

where BN, SF, and FA represent the replacement levels of bentonite, silica fume, and fly ash, respectively. Based on the experimental results, mixtures with compressive strengths below 35 MPa, such as Mix 12, would be excluded from structural applications despite their environmental benefits. This framework can support policymakers and engineers in selecting environmentally optimized concrete mixtures for structural use.

### Practical implications and future work

The findings of this study demonstrate the practical potential of ternary SCM systems for sustainable concrete production in infrastructure applications, particularly under aggressive marine exposure conditions. Optimized combinations of bentonite and silica fume improved both mechanical and environmental performance while supporting the ecological sustainability of the concrete manufacturing process.

Future studies are recommended to further investigate long-term durability through additional tests, including water absorption, sorptivity, freeze–thaw resistance, and microstructural characterization techniques such as scanning electron microscopy (SEM) and X-ray diffraction (XRD) analysis. These investigations would provide deeper insight into pore-structure evolution, capillary water transport, resistance to cyclic environmental deterioration, and the mechanisms of microstructural densification in ternary SCM systems. Prior research has demonstrated that ternary SCM mixtures can reduce porosity, water absorption, and chloride permeability by refining pores and densifying the matrix, thereby enhancing durability under aggressive service conditions^58^. The present findings are consistent with these established trends.

The present study was conducted under standard water-curing conditions. Future work should examine the influence of alternative curing methods, including steam curing, air curing, and field exposure conditions, particularly for mixtures with high fly ash content. Future studies may also investigate the modulus of elasticity of optimized ternary mixtures to further evaluate their structural applicability.

The identified Pareto gap—where compressive strength above 40 MPa and GWP reduction greater than 15% were not achieved simultaneously—highlights an important direction for future optimization. Additional improvements may be achieved through alternative SCM combinations or optimized ternary proportions. While the current Pareto frontier successfully identified two optimum mixtures for compressive strength and GWP reduction, future studies may expand this framework by integrating multi-objective optimization algorithms with additional constraints such as lifecycle cost and long-term durability indices. This approach could provide a broader and more flexible decision-making framework for sustainable concrete selection.

Future research may also extend the regression-based analysis to additional environmental indicators such as ODP, AP, and POCP to provide a more comprehensive understanding of the environmental performance of ternary SCM systems.

Regarding generalizability, the mechanical and microstructural findings are broadly transferable. However, the quantitative LCA results remain region-specific. Variations in raw material mineralogy, manufacturing efficiency, transportation scenarios, and national energy mixes may influence baseline environmental impacts and, in turn, require regional inventory calibration.

## Conclusions

This study evaluated the mechanical, durability, and environmental performance of 22 green concrete mixtures incorporating bentonite, fly ash, and silica fume. Statistical modeling (ANOVA and Tukey’s HSD) and multi-criteria optimization were applied. The main findings and engineering implications are summarized below.

1. *Statistically validated superiority of ternary systems* One-way ANOVA and Tukey’s HSD confirmed that Group III (bentonite + silica fume) outperformed Group I and Group II in all mechanical properties (*p* < 0.001), with significantly higher compressive, tensile, and flexural strengths. Mean differences reached 1.18 MPa in tensile strength and 2.27 MPa in flexural strength compared with Group II, confirming the synergistic effect of silica fume and bentonite on ITZ densification and pore refinement.
2. *Structural performance and marine durability peak* Mix 20 was identified as the best-performing mixture (*p* < 0.05), achieving a 56-day compressive strength of 54.27 MPa and a flexural strength of 6.87 MPa. Mix 17 and Mix 19 also performed well, achieving compressive strengths above 52 MPa and flexural strengths above 6.3 MPa. Mix 19 is recommended for marine exposure, having reached a seawater compressive strength of 53.07 MPa and an RCPT value of 250 Coulombs, indicating very low chloride permeability. Mix 20 is recommended for maximum structural performance applications.
3. *Environmental profiles and LCA sensitivities* Cement content remained the dominant environmental factor, with the A1–A3 stage contributing 83–95% of total GWP across all mixes. Mix 12 achieved a 50% reduction in GWP compared with the control mix, along with improvements across all other impact categories. Silica fume mixtures showed a slight increase in ODP (0.56–3.90%); sensitivity analysis demonstrated that this increase is attributable to database allocation methods rather than intrinsic material behavior.
4. *Quantitative engineering recommendations* Two application ranges are recommended: (i) for high-strength and marine infrastructure, Mixes 16–19 are suitable, providing compressive strengths between 50 and 53 MPa with moderate GWP reduction (8.7–12.3%); and (ii) for low-carbon applications, Mix 12 and bentonite-only mixes are preferred, offering stable mechanical behavior and higher carbon savings.
5. *Objective mechanical–environmental trade-off* Pareto optimization showed a clear trade-off between strength and environmental impact—no mixture achieved both compressive strength above 40 MPa and GWP reduction above 15%. This finding defines the boundary of the studied design space.

Practical Engineering Implications. Bentonite–silica fume systems are recommended for marine and chloride-rich environments due to improved durability and reduced permeability. Fly ash-based mixtures are suitable for low-carbon applications where moderate strength is acceptable. Silica fume significantly enhances ITZ densification and is recommended for high-strength structural concrete applications requiring superior mechanical performance.

Limitations and future scope. This study is limited to laboratory conditions and selected replacement ranges. Future work should include field exposure studies, shrinkage and creep evaluations, and lifecycle cost analyses to further validate the long-term performance. Additionally, exploring alternative SCM combinations may help reduce the trade-off between mechanical strength and environmental impact identified in the Pareto analysis^59–64^.

## Data Availability

The datasets generated and/or analyzed during the current study are available from the corresponding author on reasonable request.

## References

[1] Aïtcin, P. C. & Mindess, S. *Sustainability of Concrete* (CRC, 2011).

[2] WBCSD (World Business Council for Sustainable Development). *Cement industry energy and CO₂ performance: Getting the numbers right (Industry Report)* (WBCSD2023).

[3] Abutaleb, A., Kassem, E. & Nassar, A. Energy consumption and greenhouse gas emissions in the Egyptian building sector. *Energy Build.* **174**, 266–275 (2018).

[4] Hossain, M. U. & Ng, S. T. Global environmental impact of construction materials. *J. Clean. Prod.* **190**, 443–452 (2018).

[5] Rathore, Y., Raheem, J. & Raman, S. Performance evaluation of concrete incorporating Vindhyan sandstone manufactured sand and metakaolin. In * Recent Advances in Structural Engineering—Vol. 2 (RAISE 2024), Lecture Notes in Civil Engineering* (eds Kondraivendhan, B. et al.) Vol. 689. (Springer, 2025). 10.1007/978-981-96-8830-2_24

[6] Rathore, Y. & Raheem, J. Experimental investigation of concrete performance incorporating Deccan basalt manufactured sand and rice husk ash. *Nat. Acad. Sci. Lett.*10.1007/s40009-025-01703-5 (2025).

[7] Rathore, Y., Raheem, J. & Raman, S. Enhancing concrete performance using sustainable ternary blended mix with metakaolin, rice husk ash, and Deccan basalt manufactured sand. *Procedia Struct. Integr.* **71**, 401–408. 10.1016/j.prostr.2025.08.054 (2025).

[8] Raman, S., Nateriya, R. & Rathore, Y. Mechanical and durability characteristics of green concrete with recycled steel slag and ground granulated blast furnace slag. In *Recent Advances in Structural Engineering—Vol. 1 (RAISE 2024), Lecture Notes in Civil Engineering* (eds Kondraivendhan, B. et al.) (Vol. 688) (Springer, 2025). 10.1007/978-981-96-8826-5_26

[9] Kolawole, J. T., Olubanwo, A. & Akinyemi, B. Supplementary cementitious materials and their role in sustainable construction. *Constr. Build. Mater.* **242**, 118132 (2020).

[10] Teixeira, E. R., Mateus, R. & Braganca, L. Environmental life cycle assessment of concrete structures. *J. Clean. Prod.* **112**, 158–170 (2016).

[11] Yang, K. H., Jung, Y. B., Cho, M. S. & Tae, S. H. Effect of supplementary cementitious materials on concrete performance. *Constr. Build. Mater.* **75**, 105–112 (2015).

[12] Rathore, Y., Raheem, J. & Raman, S. Synergistic influence of Vindhyan sandstone manufactured sand and rice husk ash on concrete performance. *Nat. Acad. Sci. Lett.* **48**, 357–362. 10.1007/s40009-024-01484-3 (2025).

[13] He, J., Zhang, Y. & Wang, Z. Mechanical and durability properties of fly ash-based sustainable concrete. *Constr. Build. Mater.* **401**, 132411 (2024).

[14] Mekhnache, Z. et al. Enhancing cementitious materials: A detailed review of bentonite’s role in optimizing concrete performance. *J. Building Mater. Struct.* **11** (2), 107–114 (2024).

[15] Kim, J. H., Degefa, B., Jo, A., Montoya, S. A. S., Chung, C. W. & A., & Effect of heat treatment on bentonites for utilization as a supplementary cementitious material. *J. Struct. Integr. Maintenance*. **11**(1). 10.1080/24705314.2025.2609326 (2026).

[16] Amudhavalli, N. K. & Mathew, J. Effect of silica fume on strength and durability parameters of concrete. *Int. J. Eng. Sci. Emerg. Technol.* **3**(1), 28–35 (2012).

[17] Ganesan, K., Rajagopal, K. & Thangavel, K. Rice husk ash blended cement: Assessment of optimal level of replacement for strength and permeability properties of concrete. *Constr. Build. Mater.* **22**(8), 1675–1683 (2008).

[18] Lin, Y., Wang, Q. & Chen, B. Particle packing optimization in cementitious materials incorporating mineral admixtures. *Mater. Struct.* **54**, 102 (2021).

[19] Laidani, Z., Houari, H. & Bederina, M. Effect of mineral admixtures on microstructure and durability of concrete. *Constr. Build. Mater.* **206**, 135–145 (2019).

[20] Xiao, J., Li, W. & Fan, Y. Durability of concrete under sulfate and marine environments. *Constr. Build. Mater.* **415**, 135652 (2024).

[21] Al-Hammood, A. A., Frayyeh, Q. J. & Abbas, W. A. Thermally activated bentonite as a supplementary cementitious material – A review. *Eng. Technol. J.* **39**(2), 206–213 (2021).

[22] Colangelo, F., Forcina, A., Petrillo, A. & Farina, I. Life cycle assessment of concrete with recycled aggregates. *J. Clean. Prod.* **190**, 797–806 (2018).

[23] Kim, T., Tae, S. & Roh, S. Environmental impact assessment of concrete production. *J. Clean. Prod.* **112**, 122–131 (2016).

[24] Abdelaal, A., El-Hassan, H. & Shaaban, I. Environmental assessment of sustainable concrete incorporating supplementary cementitious materials. *J. Clean. Prod.* **382**, 135298 (2023).

[25] Müller, C., Knaack, U. & Klein, T. Environmental performance of building materials: A life cycle perspective. *Build. Environ.* **79**, 47–55 (2014).

[26] Akbar, A., Ali, M. & Amin, M. N. Effect of bentonite clay on the durability and mechanical properties of concrete. *Constr. Build. Mater.* **47**, 112–117 (2013).

[27] Waqas, M., Ahmad, S. & Khan, M. I. Bentonite as a supplementary cementitious material in concrete. *Materials* **14**(15), 4203 (2021).34361396

[28] Malhotra, V. M. & Mehta, P. K. *High-performance, high-volume fly ash concrete* (Supplementary Cementing Materials for Sustainable Development, 2005).

[29] Kurda, R., Silvestre, J. D. & de Brito, J. Life cycle assessment of concrete made with high volume of fly ash. *J. Clean. Prod.* **199**, 362–374 (2018).

[30] Siddique, R. & Khan, M. I. *Supplementary Cementitious Materials* (Springer, 2011).

[31] Aïtcin, P. C. *High performance concrete* (CRC, 2000).

[32] Frischknecht, R. et al. Overview and methodology: Data v2.0. Ecoinvent Report No. 1. Swiss Centre for Life Cycle Inventories. (2007).

[33] Soust-Verdaguer, B., Llatas, C. & Garcia-Martinez, A. Critical review of BIM-based LCA method to buildings. *Energy Build.* **136**, 110–120 (2017).

[34] Wang, T., Yao, W. & Zhang, D. Quantification of pozzolanic reaction degree of low-calcium supplementary cementitious materials in blended cement pastes: A selective dissolution method. *Cement Concrete Research*, **176**, 107416 (2024).

[35] Nguyen Duy, T., Viet, H., Duc, H. T., Do Anh, T. & T., & Strength development and coefficient of thermal expansion of high-strength concrete using silica fume. *Tạp Chí Khoa Học Giao Thông Vận Tải*. **76**(1), 114–123. 10.47869/tcsj.76.1.10 (2025).

[36] Mehta, P. K. & Monteiro, P. J. M. *Concrete: Microstructure, properties, and materials* 4th edn (McGraw-Hill Education, 2014).

[37] Zheng, S. et al. Influence of silica fume on the physical properties and microstructure of lightweight cements. *Adv. Cem. Res.*10.1680/jadcr.25.00076 (2025).

[38] Iqbal, M., Khan, M. I. & Siddique, R. Influence of silica fume on mechanical and durability properties of concrete. *Materials* **16**(5), 1835 (2023).36902950

[39] Soliman, M., Abdelrahman, S. & Elchalakani, M. Optimizing ultra high-performance concrete mixtures adopting the modified Andreassen approach. *Eng. Res. J.* (2024).

[40] Yang, X., Zhang, Y., Liu, Z., Wang, J. & Chen, B. Particle packing optimization and pore structure assessment of ternary cementitious system based on X-ray computed tomography and mercury intrusion porosimetry. *Constr. Build. Mater.* 134913 (2024).

[41] Jeon, S., Ryu, J. H. & Koh, T. Evaluation of pozzolanic reactivity of ferronickel slag based on particle size distribution. *International J. Concrete Struct. Materials* **19**(1), 832 (2025).

[42] Zhang, S. C., Li, K., Sun, K. M. & Wang, S. Impact of initial moisture content on the shrinkage-swelling behavior of Heishan bentonite. *KSCE J. Civ. Eng.* **26**(2), 550–555 (2022).

[43] Kroviakov, S. O. & Shymchenko, P. V. The effect of partial replacement of cement with fly ash on the strength of concrete for transportation structures and road pavements. *Sučasne Budìvnictvo Ta Arhìtektura*. **10**, 82–88. 10.31650/2786-6696-2024-10-82-88 (2024).

[44] Islam, M. M. & Islam, M. S. Strength and durability characteristics of concrete made with fly-ash blended cement. *Australian J. Struct. Eng.* **14**(3), 303–319. 10.7158/S12-037.2013.14.3 (2013).

[45] Bui, P. T., Ogawa, Y. & Kawai, K. Long-term pozzolanic reaction of fly ash in hardened cement-based paste internally activated by natural injection of saturated Ca(OH)_2_ solution. *Mater. Struct.* **51**, 144 (2018).

[46] Hemalatha, T. & Sasmal, S. Early-age strength development in fly ash blended cement composites: Investigation through chemical activation. *Magazine Concrete Res.* **71**(5), 260–270 (2019).

[47] Zaitsu, T. & Sugiyama, H. Influence of high-temperature curing in early ages on compressive strength and pozzolanic reaction of mortar mixed with fine milled fly ash. *AIJ J. Technol. Des.* **28**(70), 1101–1106 (2022).

[48] Neville, A. M. *Properties of concrete* 5th edn (Pearson Education, 2011).

[49] Ling, J., Liang, G., Zhang, J. & Yang, H. *Multiscale Analysis of Chloride Ions Transport in Seawater Geopolymers with Multiple Solid Precursors* (2026). 10.1139/cjce-2025-0392

[50] Yu, Z., Yang, J. & Yang, W. Synergistic Mechanism of Silica Fume Polystyrene Particle Concrete against Sulfate Attack and Chloride Ion Penetration. *Mater. Res. Express*. 10.1088/2053-1591/ae39b3 (2026).

[51] Zhang, B. et al. Corrosion Resistance of Fly Ash-Enhanced Cement-Based Materials in High-Chloride Gas Storage Reservoirs. *Materials*10.3390/ma19020406 (2026). 41598117 10.3390/ma19020406 PMC12843136

[52] Zhang, W. et al. Chloride binding mechanism in seawater-mixed UHPC. *Constr. Build. Mater.*10.1016/j.conbuildmat.2024.136191 (2024).

[53] Ahmad, J. et al. Partial substitution of binding material by bentonite clay (BC) in concrete: A review. *Buildings* **12**(5), 634 (2022).

[54] Ullah, Z., Rashid, M. K., Rehman, S. S. U. & Saadi, M. The impact of silica fume on the properties of high-strength concrete: Enhancing strength, workability, and durability. *Constr. Technol. Archit.* **17**, 61–70 (2025).

[55] Onyelowe, K. C. et al. Global warming potential-based life cycle assessment and optimization of the compressive strength of fly ash-silica fume concrete; environmental impact consideration. *Front. Built Environ.* **8**, 992552 (2022).

[56] Timm, J. F. G., Morales, M. F. D. & Passuello, A. Sensitivity analysis of life cycle impacts distribution methods choice applied to silica fume production. *IOP Conf. Ser. Earth Environ. Sci.* **323**(1), 012131 (2019).

[57] van den Oever, S. et al. Revisiting the challenges of ozone depletion in Life Cycle Assessment. *Qeios*10.32388/6PK4F6.2 (2023).

[58] Ji, Y., Peng, X., Tian, H. & Ding, X. Analysis of pore structure and its relationship to water transport and electrical flux in mortars incorporated with slag and silica fume. *Buildings* **15**(19), 3450 (2025).

[59] Flower, D. J. M. & Sanjayan, J. G. Greenhouse gas emissions due to concrete manufacture. *Int. J. Life Cycle Assess.* **12**(5), 282–288 (2007).

[60] Flower, D. J. M. & Sanjayan, J. G. Environmental impacts of concrete production and mitigation strategies. *J. Clean. Prod.* **112**, 147–157 (2016).

[61] Habert, G., d’Espinose de Lacaillerie, J. B. & Roussel, N. An environmental evaluation of geopolymer based concrete production. *J. Clean. Prod.* **19**(11), 1229–1238 (2011).

[62] Habibi, A., Kurda, R. & Silvestre, J. Life cycle assessment of sustainable concrete incorporating industrial byproducts. *Resour. Conserv. Recycl.* **180**, 106167 (2022).

[63] Thibodeau, C., Bataille, A. & Sie, M. Building rehabilitation life cycle assessment methodology–state of the art. *Renew. Sustain. Energy Rev.* **103**, 408–422 (2019).

[64] Van den Heede, P. & De Belie, N. Environmental impact and life cycle assessment of concrete. *Cem. Concr. Compos.* **34**(4), 431–442 (2012).

